# Exploring Deep Eutectic Solvents as Pharmaceutical Excipients: Enhancing the Solubility of Ibuprofen and Mefenamic Acid

**DOI:** 10.3390/ph17101316

**Published:** 2024-10-02

**Authors:** Mihaela-Alexandra Nica, Valentina Anuța, Cristian Andi Nicolae, Lăcrămioara Popa, Mihaela Violeta Ghica, Florentina-Iuliana Cocoș, Cristina-Elena Dinu-Pîrvu

**Affiliations:** 1 Department of Physical and Colloidal Chemistry, Faculty of Pharmacy, “Carol Davila” University of Medicine and Pharmacy, 6 Traian Vuia Str., 020956 Bucharest, Romania; mihaela.nica@drd.umfcd.ro (M.-A.N.); lacramioara.popa@umfcd.ro (L.P.); mihaela.ghica@umfcd.ro (M.V.G.); florentina.cocos@drd.umfcd.ro (F.-I.C.); cristina.dinu@umfcd.ro (C.-E.D.-P.); 2 Innovative Therapeutic Structures Research and Development Centre (InnoTher), “Carol Davila” University of Medicine and Pharmacy, 6 Traian Vuia Str., 020956 Bucharest, Romania; 3 National Institute for Research & Development in Chemistry and Petrochemistry—ICECHIM Bucharest, 202 Spl. Independentei, 060021 Bucharest, Romania; cristian.nicolae@icechim.ro

**Keywords:** deep eutectic solvents (DESs), solubility enhancement, poorly water-soluble drug, green chemistry, natural deep eutectic solvent (NADES), hydrophobic DES (HDES), ibuprofen, mefenamic acid

## Abstract

**Objectives**: The study explores the potential of various deep eutectic solvents (DESs) to serve as drug delivery systems and pharmaceutical excipients. The research focuses on two primary objectives: evaluating the ability of the selected DES systems to enhance the solubility of two poorly water-soluble model drugs (IBU and MFA), and evaluating their physicochemical properties, including density, viscosity, flow behavior, surface tension, thermal stability, and water dilution effects, to determine their suitability for pharmaceutical applications. **Methods**: A range of DES systems containing pharmaceutically acceptable constituents was explored, encompassing organic acid-based, sugar- and sugar alcohol-based, and hydrophobic systems, as well as menthol (MNT)-based DES systems with common pharmaceutical excipients. MNT-based DESs exhibited the most significant solubility enhancements. **Results**: IBU solubility reached 379.69 mg/g in MNT: PEG 400 (1:1) and 356.3 mg/g in MNT:oleic acid (1:1), while MFA solubility peaked at 17.07 mg/g in MNT:Miglyol 812^®^N (1:1). In contrast, solubility in hydrophilic DES systems was significantly lower, with choline chloride: glycerol (1:2) and arginine: glycolic acid (1:8) showing the best results. While demonstrating lower solubility compared to the MNT-based systems, sugar-based DESs exhibited increased tunability via water and glycerol addition both in terms of solubility and physicochemical properties, such as viscosity and surface tension. **Conclusions**: Our study introduces novel DES systems, expanding the repertoire of pharmaceutically acceptable DES formulations and opening new avenues for the rational design of tailored solvent systems to overcome solubility challenges and enhance drug delivery.

## 1. Introduction

In recent years, the pharmaceutical landscape has been rapidly evolving, with a growing number of complex and challenging drug molecules, including poorly soluble compounds and sensitive biologics, entering the development pipeline [1,2]. Currently, approximately 40% of approved drugs and nearly 90% of new chemicals exhibit poor solubility [2,3]. This often results in low bioavailability [4,5], hindering the drug’s ability to reach its target [6] and achieve optimal therapeutic effects.

This new generation of pharmaceuticals demands a parallel evolution in the technologies used to formulate them into safe, effective, and stable drug products [7]. While numerous approaches have been explored [8], enhancing drug solubility and overall bioavailability remains both a critical hurdle and the property with the greatest ceiling for improvement in pharmaceutical development.

Deep eutectic solvents (DESs) have emerged as a promising platform for developing novel pharmaceutical technologies to overcome this challenge [9,10].

These innovative solvents, considered a “greener” alternative to traditional ionic liquids (ILs), offer a novel and efficient method for incorporating active pharmaceutical ingredients [11,12]. DESs are formed by combining a hydrogen bond acceptor (HBA) and a hydrogen bond donor (HBD), at a specific molar ratio [13], to form liquid solutions at room temperature, significantly lowering the mixture’s melting point [14]. The term “deep” refers to the significant depression of the freezing point, which can reach up to several hundred degrees, observed in the eutectic mixture compared to the freezing points of the individual components.

This depression in melting point occurs due to strong intermolecular interactions, primarily hydrogen bonding, which stabilize the liquid state at room temperature [13]. These hydrogen bonds, with typical interaction energies ranging from −10 to −40 kJ/mol, are pivotal in DES formation [13,14,15,16,17]. The specific energy values are dependent on the nature of the chosen hydrogen bond acceptor and donor. While hydrogen bonding plays a dominant role, other non-bonded interactions including van der Waals forces and electrostatic interactions also play a crucial role. However, their energies, typically a few kJ/mol, are significantly lower than those of hydrogen bonds [18,19].

The traditional classification of DESs, proposed by Abbott et al. in 2003 [20], divides DESs into four types based on the chemistry of their constituents: Type I (quaternary ammonium salt + metal chloride), Type II (quaternary ammonium salt + metal chloride hydrate), Type III (quaternary ammonium salt + hydrogen bond donor, often an organic molecule like an amide, carboxylic acid, or polyol [21]), and Type IV (metal chloride hydrate + hydrogen bond donor). More recently, a fifth type, Type V DES, has emerged [22], distinguished by its entirely non-ionic nature, typically comprising molecular HBAs and HBDs, such as sugars [23], amino acids [24], or combinations of menthol with terpenes [25] or fatty acids [26].

Type I, II, and IV DESs often incorporate metal salts (e.g., chlorides of zinc, aluminum, or iron) [27]. While these metals have their uses, especially in industrial environments [9], they can raise toxicity concerns, especially for applications involving biological systems or drug delivery [28]. On the other hand, Type III and V DESs, by often utilizing organic salts, hydrogen bond donors, or naturally occurring molecules, tend to have more benign toxicological profiles and superior biocompatibility [29], making them particularly attractive for pharmaceutical applications [30,31]. This is further bolstered by the remarkable diversity and tunability of Type III and V DESs [29]. By carefully selecting the HBA and HBD components, researchers can improve drug solubility [32,33] or fine-tune crucial physicochemical properties like viscosity, polarity, superficial tension, or melting point, enabling the development of tailored drug delivery systems [34,35,36].

The remarkable diversity within Type III and V DESs has led to further subclassifications, each based on specific properties and applications. One particularly promising subset is Natural Deep Eutectic Solvents (NADESs), composed entirely of naturally occurring metabolites, often derived from plants, such as organic acids, alcohols, amino acids, and sugars [37]. Inspired by nature, where similar mixtures solubilize plant metabolites at concentrations exceeding their theoretical solubility in water [38], NADESs offer a compelling combination of “green” and biocompatible characteristics, low cost, and inherent stability [39,40]. These properties have positioned them as a potentially transformative tool in drug development, even earning them the moniker “solvents of the 21st century”. NADESs have already demonstrated their versatility across various fields, showing promise for applications in extraction and separation processes [41,42], electrochemistry [43,44], material science [45,46], biotechnology [47,48], or bioengineering [49]. Research into NADES applications is rapidly expanding, driven by a growing understanding of eutectic mechanisms and the potential of these bio-inspired solvents [36].

While initially recognized for their hydrophilic nature, the field of deep eutectic solvents expanded significantly with the introduction of hydrophobic DESs (HDESs) by van Osch et al. in 2015 [50]. These innovative solvents, formed using poorly water-soluble components like menthol and carboxylic acids [51], opened up new possibilities in applications requiring interactions with non-polar compounds [52,53,54] including drug delivery and formulation [55]. They are gaining attention as potential permeation enhancers in drug delivery, particularly for transdermal drug administration [56].

Further expanding the scope of DESs in drug delivery is the emergence of Therapeutic Deep Eutectic Solvents (THEDESs) [57]. In this unique category, the active pharmaceutical ingredient (API) itself serves as a component of the eutectic mixture [58]. This approach streamlines the formulation process and can substantially improve drug solubility and bioavailability [59]. A prime example is the combination of lidocaine with various NSAIDs, resulting in THEDESs that enhance drug solubility and facilitate transdermal delivery [60].

In the pharmaceutical field, preliminary studies have already demonstrated the remarkable ability of DESs to improve drug solubility and stability [61,62]. They are capable of enhancing the equilibrium solubility of poorly water-soluble drugs by disrupting their crystalline structure. The strong hydrogen bonds and van der Waals interactions within DESs interact with the drug molecules, reducing their lattice energy and facilitating their dissolution [62,63]. This mechanism is particularly effective for drugs with highly crystalline structures, where DESs can effectively lower the energy barrier for solubility [9].

Notably, DESs have been shown to enhance the solubility of various NSAIDs [64], significantly surpassing the improvements achieved with traditional cosolvent systems [65]. NADESs based on choline chloride (ChCl) and glycolic acid have demonstrated remarkable solubility enhancements for various drugs, including itraconazole, piroxicam, lidocaine, and posaconazole [66], whereas choline chloride and glycerol were used for enhancing the solubility of atenolol, carbamazepine, carvedilol, ibuprofen, ketoconazole, lamotrigine, phenothiazine, phenytoin, piroxicam, sulfamethoxazole, and tadalafil [67].

Further studies have demonstrated the versatility of DESs and NADESs in enhancing the solubility of drugs like dapsone [33], phenytoin [68], ranitidine, methylphenidate, spironolactone, or trimethoprim [64] using various combinations of HBAs and HBDs.

DESs can also significantly increase the apparent solubility of drugs in aqueous solutions [69]. At low and medium water content, DESs maintain a structured hydrogen-bonded network, creating a favorable microenvironment for solubilizing hydrophobic drugs [70,71]. This network can encapsulate drug molecules, shielding them from the bulk aqueous environment and effectively increasing their apparent solubility [72,73].

As the water content increases (above 40–50% *w*/*w*), the network transitions [73], leading to increased fluidity but still allowing for solubilization through other mechanisms, such as micelle formation, hydrotropy, and co-solvent effects [73,74,75]. This ability to modulate the solubilization environment [71] makes DESs highly versatile and effective in pharmaceutical formulations, particularly for poorly water-soluble drugs.

Beyond solubility, DESs have also exhibited the potential to enhance drug stability. Notably, the hydrolysis of aspirin was significantly slowed down in a ChCl:1,2-propanediol DES compared to water, highlighting the potential of these solvents to improve the shelf-life and efficacy of labile drugs [64]. A betaine:urea mixture was also found to substantially increase the stability of labile antibiotics [76].

What sets DESs apart from other commonly used solvents in the pharmaceutical industry is their unique combination of desirable properties. They are cost-effective, biodegradable, and generally exhibit lower toxicity compared to traditional solvents [77]. This favorable safety profile stems from their composition, often utilizing safe and naturally occurring components like sugars and organic acids [12].

Beyond their environmental benefits, DESs are remarkably easy to prepare and can be readily scaled for larger production, making them highly attractive for pharmaceutical research and development [30]. Furthermore, the absence of chemical reactions during DES formation aligns perfectly with Green Chemistry principles, minimizing environmental impact throughout their entire lifecycle [78].

Among the various components used in DES formulations, choline chloride (ChCl) stands out as the most widely employed HBA. Its affordability, widespread availability, and excellent safety profile [79], as well as its long history and well established use as a dietary supplement [80], make it a highly attractive choice for pharmaceutical use [81]. Its versatility in forming DES systems by pairing with diverse HBDs, such as carboxylic acids, amino acids, and sugars, allows tailored DES solvents with specific therapeutic applications to be obtained [82,83].

Menthol (MNT) is another promising HBA rapidly gaining traction in DES research, particularly for its role in HDES systems [51,84,85]. Its safety profile is well-established, with a high LD50 value (3000 mg/kg in rats when administered orally) [86] and Generally Recognized as Safe (GRAS) status for use in food and as a flavoring agent granted by the FDA [87]. Regulatory bodies have also granted acceptance for menthol’s use in concentrations up to 1% for oral drug products and a more substantial 16% for topical formulations, further solidifying its potential in pharmaceutical applications [88,89].

Beyond its favorable safety profile, MNT possesses a range of beneficial pharmacological properties that make it particularly well-suited for pharmaceutical formulations [90]. Its analgesic, anti-inflammatory, antiseptic, and antipruritic effects make it a valuable asset in topical formulations designed for pain relief and skin irritation [88,91]. Moreover, menthol acts as an effective permeation enhancer, improving drug absorption through mucosal membranes [92]. This is attributed to its ability to modify skin barrier properties, potentially by disrupting the lipid bilayer of the stratum corneum [92], and activating TRPM8 channels, which are known to play a role in cold sensation and pain relief [93].

Building upon the promising potential of DESs, this study aims to explore the potential of several Type III and V DESs for enhancing drug solubility and enabling the development of innovative drug delivery systems. Two widely prescribed non-steroidal anti-inflammatory drugs (NSAIDs), ibuprofen (IBU) and mefenamic acid (MFA), both classified as Biopharmaceutics Classification System Class II drugs [94], were chosen as model drugs due to their low aqueous solubility, a factor limiting their bioavailability and therapeutic efficacy [95].

IBU has a pKa of 4.45 [96] and a water solubility of approximately 40–70 mg/L at 25 °C, depending on the source and experimental conditions [64,97,98]. In contrast, MFA, with a pKa of 4.2, exhibits even lower water solubility, typically around 10–20 mg/L at 25 °C [99], making it significantly more difficult to dissolve in aqueous solutions.

Furthermore, the distinct chemical structures of these drugs—IBU as a carboxylic acid derivative and MFA as an anthranilic acid derivative [100]—offer a valuable opportunity to investigate the influence of different functional groups on drug–DES interactions.

The selection of IBU as a model drug is further strengthened by the extensive existing literature on its solubility enhancement [101,102,103,104,105], which provides a robust basis for comparison, allowing for a critical evaluation of the effectiveness and advantages of DES-based approaches over conventional methods.

In contrast, MFA remains relatively understudied, especially in the context of DES-based solubilization strategies, with no experimental data available to date, to the best of our knowledge. This presents an important opportunity to critically assess the solubilizing potential of DESs in enhancing the bioavailability of MFA, which could be particularly valuable given that its long-term oral administration is limited by severe gastrointestinal side effects such as ulcers and bleeding [106].

The present study aims to explore the potential of various Type III and Type V eutectic solvents as drug delivery systems and pharmaceutical excipients. The research focuses on two primary objectives: evaluating the ability of these DESs to enhance the solubility of two poorly water-soluble model drugs (IBU and MFA), and characterizing the physicochemical properties of the DES systems, including density, viscosity, flow behavior, surface tension, thermal stability, and water dilution effects, to determine their suitability for pharmaceutical applications.

Unlike many previously reported systems, which use toxic or non-compliant solvents, the DES systems investigated are composed of components with recognized safety profiles and established use in pharmaceutical products, with the ultimate goal to pave the way for developing innovative and patient-centric drug formulations with improved solubility, bioavailability, and therapeutic outcomes.

## 2. Results and Discussions

### 2.1. Composition and Rationale for DES Selection

The selection of components for DES formulations in this study was guided by the goal of enhancing the solubility of the selected model drugs (IBU and MFA) while maintaining biocompatibility and environmental sustainability. The principles of green chemistry played a key role in component selection, prioritizing environmentally benign, pharmaceutically acceptable, and cost-effective materials.

The study prioritized the use of environmentally friendly components, such as ChCl, organic acids, amino-acids, polyols, sugars and sugar alcohols, fatty acids, fatty acid esters, and terpenes, which align with the principles of green chemistry [107]. These components are not only capable of enhancing the pharmaceutical applicability of the DESs, but also reduce the environmental impact of their production and use [108]. The vast majority of the components have GRAS status [109] and are pharmaceutically acceptable from a regulatory point of view, and are readily available and cost-effective, making them attractive for large-scale pharmaceutical production [63].

A complete list of the DES components used in this study, along with key physicochemical properties, including, molecular weight (Mw), HBD, and HBA count, density, melting point (Tm), boiling point (Tb), octanol–water partition coefficient (logP), computed surface tension (ST) in liquid state, molar volume (MV), and topological polar surface area (TPSA), is provided in Table 1.

To further elucidate the nature of these components, we employed electrostatic potential (ESP) surface mapping of the van der Waals molecular surface using density functional theory calculations at the B3LYP/6-31G ** level of theory, using the ORCA program (version 6.0.0) [110], with the initial 3D structures being computed using the Avogadro software (version 4.2.1) [111]. The ESP surface maps, visualized in Table 1, provide insights into the electronic structure and highlight regions of positive and negative potential that are accessible for intermolecular interactions, such as hydrogen bonding [112]. Regions of high electron density, indicative of potential HBA sites, are represented in red, while areas of low electron density, suggestive of HBD regions, are depicted in blue. Green represents areas of neutral potential. The electrostatic potential increases in the order red < orange < yellow < green < blue. This detailed characterization of the components provides a foundation for understanding their individual contributions to the properties and potential applications of the resulting DES formulations.

The specific composition of all investigated DES systems is presented in Table 2. ChCl and MNT were chosen as the primary HBAs for hydrophilic and hydrophobic DES systems, respectively. However, it is crucial to acknowledge the inherent dual nature of many of the neutral compounds, including amino acids, organic acids, or sugars (Table 1). While a molecule may exhibit a dominant HBA or HBD character, it can often participate in both roles depending on the surrounding chemical environment and the nature of interacting species [113]. For this reason, studies of the newer classes of DESs, comprising non-ionic compounds, generally avoid the definitive categorization of components as solely HBAs or HBDs [114].

Recognizing that a strict categorization of components as solely HBAs or HBDs can be overly simplistic, particularly for molecules with the capacity to act as both, we have opted in this paper for a classification of the developed DES systems based on the origin (natural or synthetic) and chemical class of the primary HBD component. This approach, while still rooted in the HBD/HBA paradigm, allows for a more practical grouping of the diverse DES formulations investigated in this study, yielding four distinct categories: organic acid-based NADESs, sugar- and sugar alcohol-based NADESs, MNT-based hydrophobic NADESs, and DESs with synthetic alcohols and esters commonly used as excipients in the pharmaceutical industry.

While certain eutectics, notably G1 [115,116,117,118], G2 [119,120], L1–L3 [26,113], or D1–D2 [121,122], have been extensively researched, primarily as extraction solvents, others, including A5–A7, G3, G5–G9, L4–L11, and D3–D4, remain relatively unexplored. This gap in knowledge underscores their potential and forms the basis for their selection as promising candidates for further investigation in this study.

Our study introduces novel DES systems, expanding the repertoire of pharmaceutically acceptable DES formulations and opening new avenues for drug solubility enhancement. Unlike many previously reported systems, which use toxic or non-compliant solvents, our DES formulations are composed of components with recognized safety profiles and established use in pharmaceutical products.

**Table 1:** Physicochemical properties of selected drugs and DES components.

Comp.	Code	2D Chemical Structure	Electrostatic Potential (ESP) Surface Map	Mw (g/mol)	HBD Count	HBA Count	Density (g/cm^3^)	T_m_ (°C)	T_b_ (°C)	T_g_ (°C)	logP *	ST * (mN/m)	MV * (cm^3^)	TPSA * (Å)
Ibuprofen	IBU	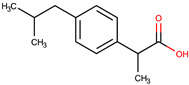	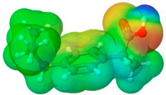	206.28	1	2	1.03 [123]	75	157 [123]	−44 [124]	3.72	38.1 ± 3.0	200.34	37.30
Mefenamic acid	MFA	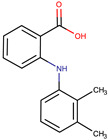	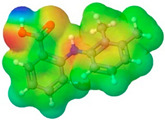	241.29	2	3	1.20 [125]	230 [126]	398.8 [125]	-	5.33	51.8 ± 3.0	200.55	49.33
Choline chloride	ChCl	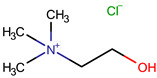	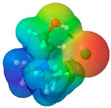	139.62	1	2	~1.205 (solid), ~1.1 (sol. 70%) [127]	302–305 (dec.) [20,128]	-	-	−5.16	-	-	20.23
(±)-menthol	MNT	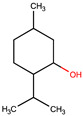	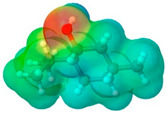	156.26	1	1	0.89 [90]	42.5 [126]	212 [129]	−54.3 [130]	3.20	29.7 ± 3.0	175.54	20.23
L-Arginine	Arg	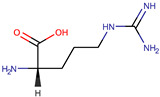	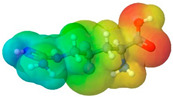	174.20	4	4	1.42 [126]	244 (dec.) [126]	-		−4.2	66.1 ± 7.0	118.72	125.22
L-Cysteine	Cys	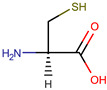	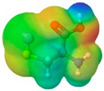	121.16	3	3	1.66 [126]	220–240 (dec.) [126]	-	-	0.23	59.0 ± 3.0	90.78	102.12
Glycolic acid	Gla	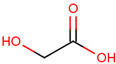	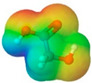	76.05	2	3	1.26 [126]	79.5 [126]	100 (dec.) [126]	-	−1.05	61.4 ± 3.0	53.68	57.53
Oxalic acid	Oxa	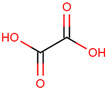	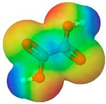	90.03	2	4	1.90 [126]	185–189.5 (dec.) [126]	- (sublimes)	-	−1.19	87.4 ± 3.0	50.80	74.60
L-lactic acid	Lac	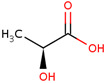	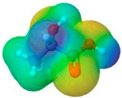	90.08	2	3	1.25 [129]	53 [126]	125–140 [131]	-	−0.70	49.8 ± 3.0	70.56	57.53
Glycerol	Gly	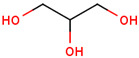	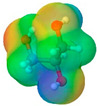	92.09	3	3	1.26 [129]	17.9–18.2 [126]	290.0 ± 0.0 [129]	−83.15 [132]	−2.32	62.0 ± 3.0	70.95	60.69
D-(+)-glucose	Glu	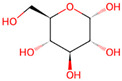	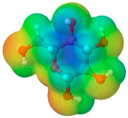	180.15	5	6	1.5620 [126]	146–165 [126,133]	-	38.87 ± 0.15 [134]	−3.17	92.1 ± 3.0	113.93	118.22
D-(−)-fructose	Fru	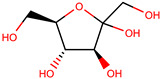	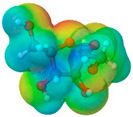	180.16	5	6	1.60 [126]	91–185 (dec.) [126]	-	16–25 [135]	−1.47	92.7 ± 3.0	106.29	110.38
D-(−)-sorbitol	Sor	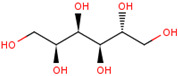	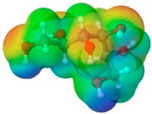	182.17	6	6	1.47 [129]	98 – 100 hydrated, 111 anhidrous [136]	295 [129]	−4.15 [132]	−4.67	99.9 ± 3.0	114.12	121.38
Xylitol	Xyl	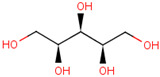	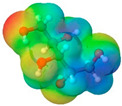	152.15	5	5	1.50 [137]	95.9 [126]	380 [126]	−23.15 [132]	−3.77	89.7 ± 3.0	99.73	101.15
Decanoic acid (capric acid)	Dec	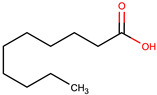	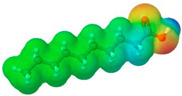	172.27	1	2	0.89 [126]	30.41 [138]	270 [126]	-	3.97	33.2 ± 3.0	188.23	37.30
Oleic acid	OLA	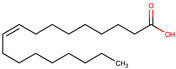	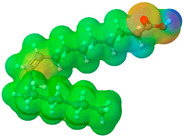	282.47	1	2	0.89 [126]	13–14 [126]	360 [139]	-	7.7	33.9 ± 3.0	313.90	37.30
Medium chain tryglicerides (Miglyol^®^ 812)	MCT	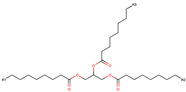 R1, R2, R3: -H, -C_2_H_5_	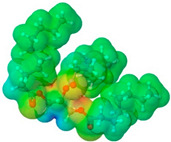	498.75	0	6	0.93–0.96 [139]	6.0 [139]	-	-	10.39	34.7 ± 3.0	518.05	78.90
(R)-(+)-limonene	Lim	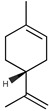	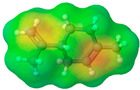	136.24	0	0	0.84 [126]	−96.9 [129]	195 [129]	-	4.45	25.9 ± 3.0	163.26	0.00
Polyethylene glycol 400	PEG 400	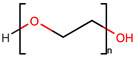	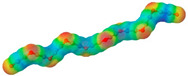	380–420	2	10	1.12 [126]	5.8 [140]	240–250 [126]	−81 [124]	−4.02	40.8 ± 3.0	371.57	114.30
Isoporpyl miristate	IPM	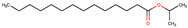	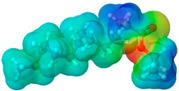	270.46	0	2	0.85 [126]	3.0 [126]	193 [126]	-	7.43	29.7 ± 3.0	313.01	26.30
Propylene glycol	PG	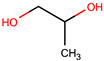	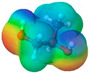	76.10	2	2	1.04 [126]	−60 [126]	187.3 [126]	−101.15 [141]	−1.34	38.0 ± 3.0	73.44	40.46

*—computed using ACD/Labs Percepta Platform-PhysChem Profiler Module, release 2023.2.4 (Advanced Chemistry Development Inc., Toronto, ON, Canada).

**Table 2:** Composition, density, and solubility of IBU and MFA in various experimental DES systems.

Code	Components				Density (g/cm^3^)	Solubility (mg/g)	
	1	2	3	Molar Ratio		IBU	MF
A1	ChCl	Gla	-	1:1	1.2117 ± 0.0314	8.610 ± 0.067	0.832 ± 0.033
A2	ChCl	Gla	-	1:2	1.2597 ± 0.0218	3.992 ± 0.043	0.481 ± 0.043
A3	ChCl	Oxa	-	1:1	1.2814 ± 0.0222	1.512 ± 0.016	0.158 ± 0.016
A4	ChCl	Lac	-	1:1	1.2366 ± 0.0748	8.463 ± 0.011	0.104 ± 0.006
A5	Arg	Gla	-	1:8	1.4479 ± 0.0876	48.695 ± 0.112	1.861 ± 0.205
A6	Arg	Lac	-	1:8	1.2372 ± 0.0428	3.850 ± 0.005	0.087 ± 0.005
A7	Cys	Gla	-	1:4	1.4821 ± 0.0641	16.827 ± 0.020	0.341 ± 0.027
G1	ChCl	Gly	-	1:2	1.1821 ± 0.0920	132.330 ± 0.081	1.344 ± 0.148
G2	ChCl	Glu	-	1:1	1.3082 ± 0.0565	0.543 ± 0.01	0.106 ± 0.003
G3	ChCl	Sor	-	1:1	1.2792 ± 0.0774	2.758 ± 0.005	0.082 ± 0.002
G4	ChCl	Xyl	-	1:1	1.2718 ± 0.0220	1.382 ± 0.005	0.083 ± 0.002
G5	ChCl	Sor	Gly	2:1:1	1.2330 ± 0.0853	54.619 ± 0.110	1.217 ± 0.097
G6	ChCl	Glu	Gly	2:1:1	1.2409 ± 0.0322	12.286 ± 0.028	0.396 ± 0.028
G7	ChCl	Xyl	Gly	2:1:1	1.2241 ± 0.0529	32.423 ± 0.057	0.812 ± 0.049
G8	ChCl	Glu	water	1:1:1	1.2829 ± 0.0998	38.084 ± 0.160	2.006 ± 0.100
G9	ChCl	Sor	water	1:1:1	1.2632 ± 0.0983	17.958 ± 0.055	0.919 ± 0.092
G10	ChCl	Xyl	water	1:1:1	1.2582 ± 0.0326	7.018 ± 0.148	1.650 ± 0.165
G11	ChCl	Fru	water	1:1:1	1.2982 ± 0.0673	48.944 ± 0.310	3.445 ± 0.344
G12	Glu	Lac	-	1:5	1.2773 ± 0.0552	49.098 ± 0.004	0.066 ± 0.003
L1	MNT	Dec	-	1:1	0.8924 ± 0.0694	330.347 ± 33.035	4.230 ± 0.169
L2	MNT	Dec	-	2:1	0.8872 ± 0.0383	302.445 ± 9.073	3.579 ± 0.394
L3	MNT	Dec	-	1:2	0.8972 ± 0.062	356.299 ± 24.941	4.469 ± 0.447
L4	MNT	Ola	-	1:1	0.8887 ± 0.0538	279.169 ± 11.167	3.794 ± 0.190
L5	MNT	Ola	-	1:2	0.884 ± 0.0229	319.254 ± 9.578	3.814 ± 0.267
L6	MNT	Ola	-	2:1	0.8813 ± 0.0457	272.940 ± 10.918	2.836 ± 0.312
L7	MNT	MCT	-	1:1	0.8824 ± 0.0686	225.197 ± 13.512	17.068 ± 1.707
L8	MNT	MCT	-	1:2	0.8803 ± 0.0380	234.432 ± 11.722	13.208 ± 0.528
L9	MNT	Lim	-	1:1	0.8760 ± 0.0606	263.229 ± 7.897	4.854 ± 0.146
L10	MNT	Lim	-	1:2	0.8732 ± 0.0302	232.844 ± 20.956	4.620 ± 0.462
L11	MNT	Lim	-	2:1	0.8816 ± 0.0533	275.228 ± 11.009	4.579 ± 0.458
D1	ChCl	PG	-	1:2	1.1294 ± 0.0586	204.520 ± 8.181	1.060 ± 0.042
D2	ChCl	PG	-	1:3	1.0623 ± 0.0367	196.468 ± 17.682	7.848 ± 0.628
D3	MNT	PEG 400	-	1:1	1.0293 ± 0.0178	379.685 ± 22.781	3.838 ± 0.115
D4	MNT	IPM	-	1:1	0.8672 ± 0.0225	269.091 ± 26.909	5.617 ± 0.506
W	Water	-	-	-	-	0.056 ± 0.003	0.042 ± 0.002

#### 2.1.1. Organic Acids-Based NADESs

This class encompasses NADESs formulated using various organic acids, including oxalic acid (Oxa), glycolic acid (Gla), and lactic acid (Lac) (A1–A4) as HBDs, while ChCl was used as the primary HBA.

The selection of the organic acids as HBDs for NADES formulations was guided by their diverse functionalities and established applications in pharmaceutical and cosmetic industries, together with their capability to produce liquid, stable DES systems resistant to crystallization.

Oxa, a strong HBD with two carboxyl groups, exhibits potent metal-chelating properties. This chelation contributes to antioxidant capacity and formulation stability by inhibiting metal-catalyzed degradation [142]. Furthermore, the chelation of essential metal ions from microorganisms can disrupt their cellular processes, contributing to the antimicrobial efficacy [143,144]. A study by Radosevic et al., on antimicrobial, cytotoxic, and antioxidant properties of different NADESs, identified the oxalic acid-based systems as very potent antimicrobials against most of the common bacterial strains [145].

Gla, the smallest alpha hydroxy acid (AHA), is well known for its keratolytic properties, making it effective in treating skin disorders like acne, aging, and keratoses [146]. Its small molecular size allows it to penetrate the skin effectively, facilitating the delivery of other active ingredients [147].

Lac, a naturally occurring AHA, is a versatile organic compound with significant applications in the pharmaceutical industry, due to its capacity to regulate skin pH and enhance the penetration of active ingredients in transdermal drug delivery systems [147,148]. Moreover, its application extends to biodegradable materials for wound healing, showcasing its diverse therapeutic potential [149,150].

To further diversify our NADES formulations and explore the impact of HBA structure on drug solubility, we incorporated amino acids as alternative HBAs. Specifically, we paired Arg and Cys with Gla and Lac (A5–A7). Previous research has demonstrated Arg ability to enhance lidocaine solubility through eutectic systems with organic acids like glutamic, oxalic, and tartaric acid, highlighting its potential as a solubility enhancer [151]. Furthermore, a Cys:Lac (1:8) DES system has been investigated for its iodine removal capacity, suggesting the potential of this pairing in other applications [152,153]. Our study aims to extend these findings, exploring new perspectives on Arg and Cys-based NADESs for solubilizing poorly soluble drugs.

#### 2.1.2. Sugar- and Sugar Alcohol-Based NADESs

Recognizing the remarkable ability of sugars and sugar alcohols to form eutectic mixtures and enhance the solubility of various compounds [154], we investigated a series of NADESs comprising ChCl as the HBA and glucose (Glu), sorbitol (Sor), or xylitol (Xy)l as HBDs (G1–G4). To further modulate and fine-tune the physicochemical properties and drug-solubilizing capacity of these NADESs, optimizing them for better drug formulation outcomes, we also incorporated water and glycerol (Gly) as ternary components [71,155].

Furthermore, incorporating sugars and sugar alcohols in NADES formulations offers the potential for taste-masking, a significant advantage in developing palatable oral dosage forms. This is particularly relevant for pediatric and geriatric patients who may have difficulty tolerating bitter or unpleasant-tasting medications.

#### 2.1.3. Hydrophobic MNT-Based NADESs (HNADESs)

Hydrophobic NADESs, primarily based on MNT as the HBA, are emerging as promising candidates for both topical and transdermal drug delivery [156], as well as novel antimicrobial agents [157]. This dual potential stems from their unique ability to interact with and disrupt lipid-rich biological barriers, whether it be the skin’s stratum corneum or the cell membranes of bacteria.

Their properties make them ideal solvents for a wide range of hydrophobic drugs [52], enhancing their solubility and creating a stronger concentration gradient that drives drug absorption through the skin. Furthermore, HNADESs can directly interact with the lipid bilayers of the stratum corneum, the skin’s outermost layer [158]. This interaction transiently loosens these tightly packed structures, allowing drugs to permeate more easily [159].

Beyond their role in drug delivery, HNADESs are gaining increasing attention for their antimicrobial properties [160]. Just as they can interact with the lipid bilayers of the skin, HNADESs can also penetrate and disrupt the lipidic cell membranes of bacteria, compromising their integrity and leading to cell death [157]. This mechanism of action has proven effective against both Gram-positive and Gram-negative bacteria, suggesting broad-spectrum activity. Importantly, because this mechanism differs from that of traditional antibiotics, HDESs may offer a way to circumvent the growing problem of antibiotic resistance [157].

To explore the potential of MNT-based NADESs, a series of formulations (L1–L11) were designed, incorporating decanoic acid (Dec), oleic acid (Ola), medium-chain triglycerides (MCTs), and limonene (Lim). MNT, with its known permeation-enhancing properties, was chosen as the HBA for its ability to facilitate drug transport across the skin barrier. The selection of HBDs with varying polarity and viscosity aimed to create a diverse range of hydrophobic environments, allowing for the fine-tuning of NADES properties to optimize their suitability for specific applications.

Dec, a medium-chain fatty acid, was selected in conjunction with MNT, due to the extensive previous research of this combination as both antimicrobial agent [90] and effective extraction solvent for hydrophobic molecules [161,162]. It is important to note that utilizing longer-chain saturated fatty acids with MNT is often impractical due to their higher melting points, which are near room temperature for lauric acid and even higher for those with longer chains [163].

Ola, a prominent fatty acid in microemulsion formulations [164], presents a valuable opportunity to further develop HNADES-based nanoparticulate pharmaceutical systems, with the HDES system used as the oil component [165].

Mygliol^®^ 812N, is a medium-chain triglyceride (MCT) oil, derived from fractionated coconut or palm kernel oil, consisting mainly of triglycerides formed by caprylic (C8) and capric (C10) fatty acids in an approximate 70:30 mass ratio, widely used in the pharmaceutical industry for its solubilizing, emollient and penetration-enhancing properties [166].

Lim, a non-polar monoterpene hydrocarbon, is less capable of traditional hydrogen bonding due to its lack of polar functional groups (Table 1). However, it can still form stable eutectic mixtures with MNT, mainly through van der Waals forces, steric effects, and hydrophobic interactions [167]. Lim’s versatility stems from its multifaceted properties, including solubility enhancement, skin penetration, anti-inflammatory, antimicrobial, and potential anticancer activities [168,169].

By systematically varying the HBDs in our HNADES formulations, we aim to establish a comprehensive understanding of the relationship between NADES composition, physicochemical properties, and their suitability for targeted pharmaceutical and biomedical applications.

#### 2.1.4. DESs with Synthetic Common Pharmaceutical Excipients

To bridge the gap between DES research and pharmaceutical applications, we investigated DES formulated using synthetic common pharmaceutical excipients with recognized solubilizing and permeation-enhancing properties, like polyethylene glycol 400 (PEG 400), propylene glycol (PG), and isopropyl myristate (IPM) (D1–D4). These DESs offer potential advantages in terms of regulatory acceptance and compatibility with existing pharmaceutical manufacturing processes.

### 2.2. Drug Solubility Evaluation

**Figure 1:**
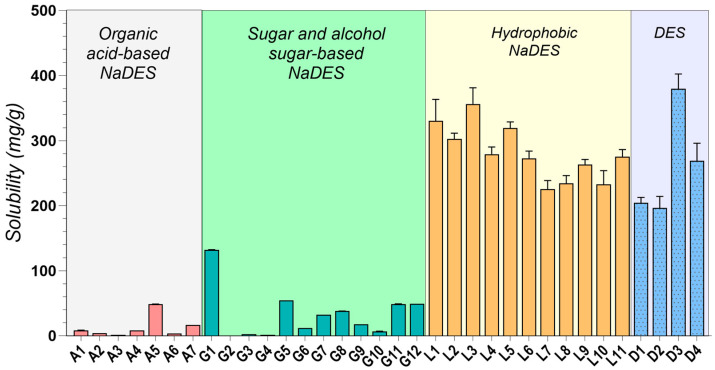
Solubility of IBU in various DESs and NADESs systems.

As depicted in Figure 1, MNT-based DESs (L1–L11, D3, D4) consistently exhibited the highest IBU solubility, exceeding 200 mg/g in all cases. This observation strongly suggests a favorable interaction between IBU and the hydrophobic microenvironment created by MNT. This interaction is likely driven by a combination of weak hydrogen bonding, van der Waals forces, and other non-polar interactions, which effectively disrupt the crystalline structure of IBU [50,170,171].

Among the hydrophobic MNT-based NADESs, MNT:Dec (L3) achieved the highest IBU solubility at 356.3 ± 24.941 mg/g, followed by MNT:Ola (L5) at 319.25 ± 9.578 mg/g (L3–L5, *p* < 0.0001). The strong hydrophobic interactions between MNT and the fatty acids likely contribute to the superior performance of these systems [84]. It is important to note that IBU itself can act as a hydrogen bond donor, potentially forming a THEDES with MNT, as confirmed by previous studies [59,172].

Additionally, it was observed that increasing the concentration of HBDs such as fatty acids and their esters (e.g., Dec, Ola, and MCT) correspondingly increased the solubility of IBU in these MNT-based NADESs, further suggesting that the solubility of IBU is closely linked to the hydrophobic interactions within these systems, as already reported in the literature [167]. The effect was significant for Dec (L1–L3, *p* = 0.0002), as well as for Ola (L5–L6, *p* < 0.0001), while minimal for MCT (L7–L8, *p* = 0.1760).

The combination of MNT and PEG 400 in a 1:1 molar ratio (D3) yielded a remarkable IBU solubility of 379.69 ± 22.718 mg/g, significantly higher than the maximum solubility achieved through HNADESs (L3–D3, *p* = 0.0008). This finding highlights the synergistic effect of PEG 400’s solubilizing capacity, attributed to its hydrogen bonding ability, and MNT’s hydrophobic character.

Our investigation aligns with existing literature, while exploring novel, pharmaceutically acceptable systems. As observed in previous studies, MNT-based DESs consistently yielded the highest solubility improvements. For instance, Lázaro-Rangel et al. reported a remarkable 338 mg/mL IBU solubility using a MNT:malonic acid 1:4 DES system [173], while Phaechamud et al. achieved a maximum IBU solubility of 282 mg/mL with a MNT:camphor DES [174]. Our findings using MNT combined with Dec, Ola, MCT, Lim, PG, PEG 400, and IPM further solidify the efficacy of menthol-based systems, achieving consistent solubility values greater than 200 mg/mL, up to a maximum of 379.69 ± 22.718 mg/g, expanding the repertoire of pharmaceutically acceptable MNT-based systems.

Amongst the hydrophilic NADESs, ChCl:Gly (1:2) (G1) exhibited remarkably high IBU solubility (132.33 ± 0.081 mg/g), indicating its potential as a more biocompatible alternative to MNT-based systems. While binary sugar-based NADESs (G2–G4) showed relatively low IBU solubility (0.54–2.76 mg/g), incorporating glycerol or water as a third component (G5–G11) significantly enhanced it (G3–G5, *p* = 0.0004). This observation underscores the effectiveness of ternary mixtures as a viable strategy for modulating drug solubility in NADESs. The low IBU solubility in sugar-based systems aligns with the work of Lomba et al., who reported achieving a maximum solubility of only 0.442 mg/mL using ChCl:xylitol (1:2)-based systems [175].

The observed solubility enhancement in ternary systems can be attributed to the disruption of the dense hydrogen bonding network characteristic of sugar-based NADESs [71]. Binary mixtures, characterized by strong intermolecular interactions between ChCl and the sugar component (e.g., Glu, Fru, Sor, and Xyl), typically form tightly packed structures [176]. This dense packing limits the available space within the NADES, hindering the accommodation and dissolution of drug molecules.

However, the introduction of water or glycerol as a third component disrupts these pre-existing hydrogen bonds between ChCl and the sugar, effectively “loosening” the tightly packed network. This disruption creates “free space” within the NADES, allowing for a greater accommodation and enhanced solubility of drug molecules [71].

Furthermore, the choice of HBD significantly influenced IBU solubility in ChCl-based NADESs. For instance, Gla (A1, A2) led to higher solubility compared to oxalic acid (A3) or lactic acid (A4). The molar ratio effect (A1 vs. A2) is also consistent, with higher Gla proportions decreasing solubility (A1–A2, *p* < 0.0001), possibly due to competition effects [10].

The use of amino acids, which possess multiple HBD and HBA functional groups, opens up interesting possibilities for DES design. Formulations like A5, comprising Arg and Gla (1:8), are capable of forming strong hydrogen bonds between the guanidine group of Arg and Gla, creating a highly interactive environment that yielded a notable IBU solubility of 48.7 ± 0.112 mg/g. This finding suggests that exploring amino acid-based NADESs could be a promising avenue for further research. This aligns with the work of Pedro et al. [177], who demonstrated the potential of an Arg-based system for enhancing IBU solubility (up to 46.92 mg/mL) and its subsequent use in developing alginate hydrogels for transdermal drug delivery.

Several other studies have reported promising IBU solubility enhancements using DESs [64,178]. However, many rely on components unsuitable for pharmaceutical applications. For example, Lu et al. [64] demonstrated high IBU solubility in a (TPAB) and PG DES system. However, the inherent toxicity of TPAB precludes its use in pharmaceutical formulations. Similarly, while a recent study highlighted the efficacy of polyol-based HBDs like diethylene glycol and ethylene glycol for IBU solubilization [178], their use is prohibited by regulatory agencies, and their presence in any medicinal formulation is considered a serious contamination due to toxicity concerns [179].

**Figure 2:**
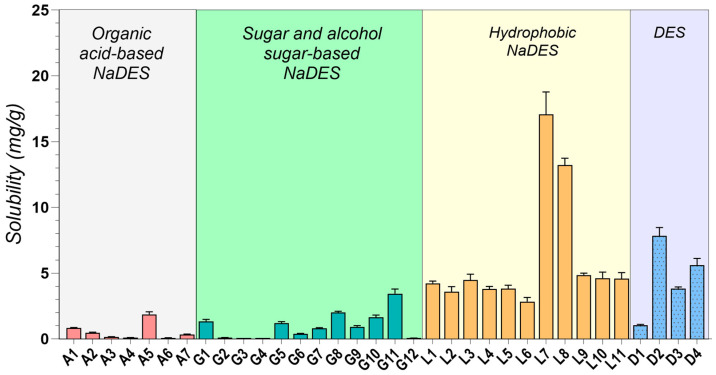
Solubility of MFA in various DESs and NADESs systems.

In contrast to IBU, MFA exhibited lower solubility across most tested DESs, likely due to its potentially different intermolecular interactions and physicochemical properties (Figure 2). MFA possesses a more complex molecular structure than IBU, featuring two large, planar aromatic rings and an amine group (Table 1). This complexity can contribute to steric hindrance, potentially limiting the molecule’s ability to interact effectively with DES components. Additionally, the proximity of the amine and carboxylic acid groups in MFA allows for intramolecular hydrogen bonding [180], reducing the availability of the -COOH group for interactions with the DES. Furthermore, the significantly higher melting point of MFA compared to IBU reflects stronger intermolecular forces in its solid state. These stronger forces necessitate greater energy to dissolve MF, likely contributing to its lower solubility in the tested DES.

Similar to IBU, hydrophobic MNT-based NADESs demonstrated the highest solubility for MF, with MNT: MCT systems (L7, L8) peaking at 17.068 ± 1.707 mg/g. However, unlike IBU, the addition of glycerol or water to sugar-based NADESs did not consistently improve MFA solubility, suggesting that optimizing MFA solubility might require more tailored approaches. MFA’s larger and more complex structure, compared to IBU, might experience greater steric hindrance within the ChCl-based eutectics, limiting its solubility.

To our knowledge, this study provides the first experimental solubility data for MFA in DESs. Despite the observed lower solubility compared to IBU, the enhancements achieved using DESs significantly surpass the values obtained using conventional techniques like cyclodextrin inclusion [181], nano crystallization [182], or solid dispersions [183], which typically yielded only a 2–10-fold increase. This highlights the potential of DESs as a promising avenue for improving the solubility and, consequently, the formulation of poorly soluble drugs like MFA.

The significant solubility improvements observed with NADESs, particularly for IBU, highlight their superior efficiency and potential as innovative solubilizing agents compared to traditional techniques like cosolvents or surfactants [31]. The solubility of IBU in water was only 0.056 ± 0.0032 µg/mL, whereas in the best-performing NADESs (D3), it reached 379.69 ± 22.718 mg/g, representing an astounding over 6700-fold increase. Equally impressive is the 400-fold solubility enhancement of MFA by the L7 system compared to its solubility in water (0.042 ± 0.002 mg/g).

### 2.3. Density Evaluation

The density data reveal a clear distinction between hydrophobic and hydrophilic DESs systems (Table 1). As expected, and consistent with existing literature [54], hydrophobic DESs, (L1–L11, D4), exhibit significantly lower densities, below 0.9 g/cm^3^, compared to their hydrophilic counterparts. This can be attributed to the inherent nature of their components, menthol, fatty acids, and terpenes, commonly employed in hydrophobic DESs, which possess significant nonpolar characters and tend to experience weaker intermolecular forces (like van der Waals forces) compared to the stronger hydrogen bonding prevalent in hydrophilic DESs [120]. This difference in intermolecular forces can lead to less compact packing in hydrophobic DESs, contributing to their lower densities.

On the other hand, hydrophilic NADESs typically display densities above 1.2 g/cm^3^ (Table 1). This property is largely influenced by the selection of the HBA and HBD, as hydrophilic components such as choline chloride, sugars, and polyols engage in extensive hydrogen bonding networks, which promote denser molecular arrangements. The choice of HBA and HBD is crucial, as their interplay directly influences the system’s density, by affecting the balance between molecular volume and intermolecular forces, ultimately affecting the free volume within the liquid—the spaces or “holes” between molecules [184]. This tunability makes DESs suitable for a wide range of applications [29,154].

While density itself does not dictate viscosity, the molecular characteristics that influence density can also impact a DESs’s viscosity. For instance, the weaker intermolecular forces and less compact packing often observed in hydrophobic DESs, contributing to their lower densities, can also lead to lower viscosities. Conversely, the stronger hydrogen bonding and denser packing in hydrophilic NADESs, resulting in higher densities, can contribute to the molecules to have more friction and to the system to exert higher viscosity [185,186].

### 2.4. Additional Physicochemical Evaluation

Twelve deep eutectic solvent systems (A1, A2, A3, A5, G1, G3, G5, G9, L5, L7, D3, and D4) were strategically chosen for further physicochemical evaluation based on a selection process based on a combination of solubility performance, pharmaceutical benefits, and patient acceptability. While a wider range of DESs systems were initially screened, only those demonstrating a favorable profile across these three key criteria were selected.

MNT-based systems like L5, L7, D3, and D4 were primarily chosen based on their high solubilizing performance for both IBU and MFA. Similarly, high drug solubility was the primary reason for choosing hydrophilic NADESs like A5 and G1.

Beyond simple solubility enhancement, the intrinsic pharmaceutical potential of some DESs systems played a pivotal role in their selection. Organic acid-based systems like A1, A2, and A3, known for their antimicrobial and keratolytic properties, hold significant promise for dermatological applications, making them particularly attractive for further development.

Sorbitol-based systems (G3, G5, and G9) were also selected for additional studies, due to a combination of favorable attributes. Their potential for taste-masking, a significant advantage in formulating palatable oral dosage forms, combined with their solubilizing potential and tunability via water and glycerol addition distinguished them as promising candidates for advanced study.

Importantly, patient acceptability served as a critical filter in the selection process. Despite demonstrating high solubilizing capacity, DESs systems based on Dec were excluded due to their unfavorable organoleptic properties. The unpleasant, “goat-like” odor of Dec would likely lead to poor patient acceptance, making it unsuitable for pharmaceutical applications.

#### 2.4.1. Rheological Study

The rheological properties of DESs, particularly viscosity, are pivotal for their successful implementation in drug delivery systems [12]. Viscosity directly influences drug diffusion and release kinetics, making it a critical design parameter for various pharmaceutical applications [64,187]. Low-viscosity DESs, desirable for immediate-release formulations, promote rapid drug diffusion and absorption [188]. This property also benefits manufacturing processes, as viscosities below 100 mPa·s are generally preferred for their ease of handling, transferring, mixing, and filling [189].

Conversely, high-viscosity DESs are advantageous for controlled drug release over extended periods, particularly in topical and mucosal drug delivery where enhanced residence time and improved drug permeation are desired [190]. The development of eutectogels, DESs with gel-like properties achieved by incorporating gelling agents, further underscores the importance of rheological control in these systems [191].

The viscosity of ionic DESs (such as those comprising ChCl as HBA) can be effectively explained using hole theory [192]. According to this theory, the viscosity of ionic liquids is influenced by the presence of “holes” or voids in the liquid structure, representing regions of free volume within the solvent that facilitate molecular movement. They are highly dependent on the nature (specific hydrogen bonding interactions) and size (through steric hindrance) of the species involved in DESs formation [184]. Thus, in highly structured ionic DESs with extensive hydrogen bonding networks, ion mobility is restricted, leading to higher viscosity. Conversely, weaker hydrogen bonding or the introduction of plasticizers disrupts this network, increasing free volume 192].

Hydrophobic DESs, typically composed of menthol, fatty acids, or terpenes, primarily rely on van der Waals interactions [114]. These weaker interactions generally result in lower viscosities compared to their ionic counterparts.

This interplay between molecular structure, free volume, and viscosity allows for the design of DESs with tunable rheological properties. By adjusting the composition and molar ratio of DESs components, researchers can achieve viscosities ranging from low-viscosity systems for rapid drug release to more viscous formulations for controlled, sustained delivery. This fine-tuning of viscosity enables optimized drug release profiles and therapeutic outcomes for a wide range of applications.

**Figure 3:**
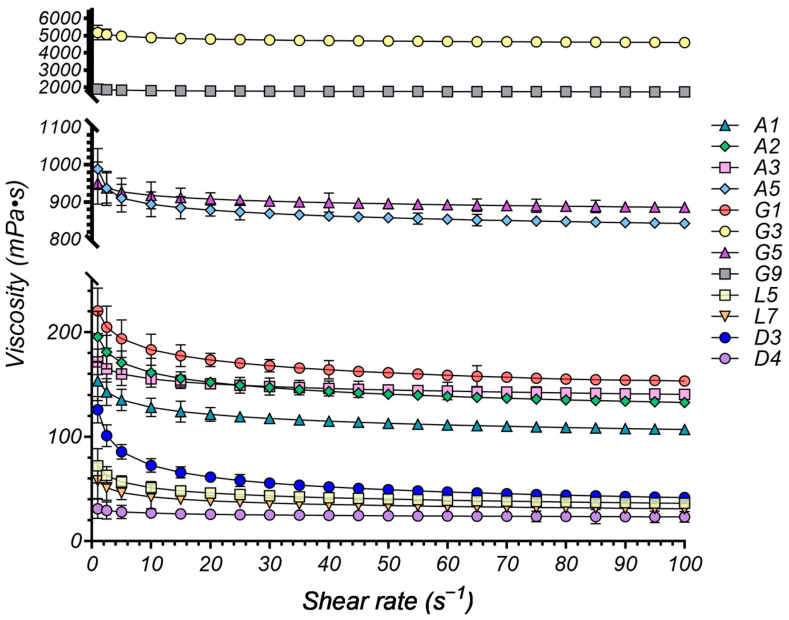
Viscosity versus shear rate for various DESs systems at 37 °C.

To evaluate the flow behavior of the experimental DESs systems, we conducted rheological measurements at 37 °C. The relationship between viscosity (η, mPa·s) and shear rate ($\dot{\gamma }$, s^−1^) was examined to determine the flow properties of the DESs. Figure 3 presents several representative rheograms obtained, illustrating the viscosity profiles as a function of shear rate.

The rheological behavior of the DESs systems was successfully modeled using the Power-law model, as evidenced by the high R^2^ values (all above 0.9968), indicating a good fit between the model and the experimental data (Table 3).

The consistency index (K) values vary significantly across the different DESs compositions, reflecting differences in their overall resistance to flow, with higher K values indicating a more viscous system.

The organic acid-based NADESs systems generally exhibit relatively low viscosity, with K values ranging from 0.154 to 0.196 Pa·s^n^, suggesting good fluidity, leading to ease of handling and potential for use in formulations requiring low viscosity, such as oral liquids or sprays. The choice of HBD appears to have a minor impact on viscosity within this group, as seen in the similar K values for A1 (Gla) and A3 (Oxa).

A5, consisting of Arg: Gla (1:8) is, however, an exception, and deviates significantly with a much higher K value (0.949 Pa·s^n^). This can be attributed to the presence of Arg, an amino acid with multiple hydrogen bonding sites, leading to a more structured and viscous DESs. Despite its relatively high viscosity, A5 exhibits good solubility enhancement, likely due to Arg’s ability to form multiple hydrogen bonds with both the drug and Gla.

The sugar-based DESs systems exhibited the highest viscosity values among the studied formulations, as indicated by their high consistency index (K) values. The binary G3 DESs exhibits the highest K value (5.191 Pa·s^n^), suggesting a high viscosity, compared to the other formulations, that could be attributed to the strong hydrogen bonding network formed by Sor, and is associated with the lowest solubility for the model drugs.

The addition of Gly (G5) dramatically reduces viscosity to 0.951 Pa·s^n^, highlighting its effectiveness as a plasticizer in disrupting the structured network of Sor and enhancing drug–solvent interactions [193], enhancing the fluidity and solubilizing capacity of the DESs.

While less pronounced than glycerol, the addition of water (G9) also reduces viscosity compared to G3, indicating its plasticizing effect, albeit weaker.

The hydrophobic systems based on MNT as the HBA, exhibit the lowest viscosity among all categories, with K values between 0.031 (D4) and 0.072 (L5), associated with the highest solubility enhancement. This suggests that the weaker hydrogen bonding associated with MNT, combined with the hydrophobic nature of the HBDs, favors drug solubilization.

The rheological behavior of DESs systems is well-documented to be influenced by the nature of the hydrogen bond donor (HBD) and hydrogen bond acceptor (HBA). While several studies have reported Newtonian behavior in DESs systems [194,195,196], others emphasize the tunability of these systems. Depending on factors such as temperature, the nature of the components, and their ratios, DESs can exhibit either Newtonian or shear-thinning behavior [197,198,199], with the optimized systems tending to exhibit Newtonian characteristics [197].

Analyzing the flow behavior index (n) of our experimental systems provides further insights into the rheological properties of the DESs. The vast majority of the evaluated systems (A1, A2, A3, A5, G1, G3, G5, G9, and D4) exhibit near-Newtonian behavior with n values exceeding 0.9159, closely approaching the ideal Newtonian value of one.

In contrast, L5 and L7, containing Ola and MCTs, display a slight shear-thinning tendency (n = 0.8513 and 0.8651, respectively). This suggests their viscosity decreases slightly under increasing shear stress, probably caused by the long hydrocarbon chains in their structure. D3, however, exhibits a pronounced shear-thinning behavior (n = 0.7603), indicating a more significant decrease in viscosity as shear stress rises. This deviation from Newtonian behavior in L5, L7, and particularly D3, likely arises from the molecular structures and interactions of their specific components.

The deviation observed in the PEG 400-based DESs (D3) can be attributed to the polymeric nature of PEG 400. Unlike the small molecules in other DESs components, polymers introduce chain entanglement that contributes to non-Newtonian behavior [200], leading to shear-thinning (n = 0.7603).

#### 2.4.2. Surface Properties

Surface tension, a fundamental property of liquids, plays a crucial role in pharmaceutical sciences, particularly in drug formulation and delivery 95]. It represents the cohesive forces within a liquid that cause its surface to contract and resist external forces [201]. Surface tension influences a solvent’s ability to wet a solid drug particle, a critical factor in dissolution and ultimately, drug bioavailability. Lower surface tension generally improves wettability and enhances solubility. Surface tension affects how formulations interact with biological membranes, impacting drug absorption and distribution within the body. 

**Figure 4:**
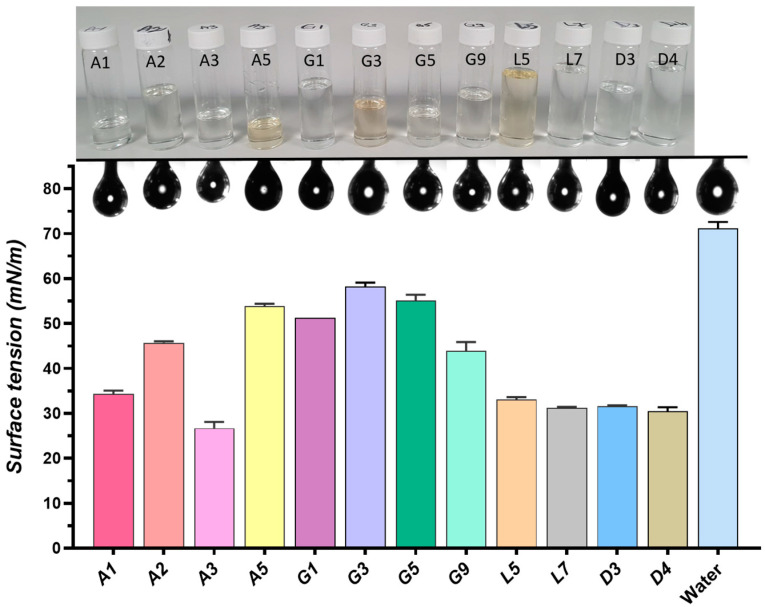
Surface tension variation of different DESs systems, measured in mN/m. The bar graph shows the surface tension values for each DESs system, while the images above the bars represent the pendant drops measured during goniometric analysis. The vials displayed above correspond to each DESss system, illustrating their appearance in liquid form.

A graphical representation of the surface tension variation for the analyzed DESs is provided in Figure 4, together with the pendant drops measured during goniometric analysis.

The organic acid-based NADESs (A1–A5) exhibit moderate surface tension values (34–53 mN/m), suggesting a balance between the hydrogen bonding capacity of the components and their overall cohesive forces.

The sugar-and sugar alcohol-based NADESs (G1, G3, G5, G9) generally have higher surface tension values (43–58 mN/m) compared to the organic acid-based NADESs, likely due to the strong hydrogen bonding network formed by the sugars, which create more cohesive systems with higher resistance to surface expansion. The addition of Gly in G5 and water in G9 significantly reduces surface tension compared to the binary G3 system composed of ChCl and Sor (1:1), highlighting their ability to disrupt the structured network of sugars. This reduction in surface tension could enhance drug solubility by improving the DES’s ability to interact with and dissolve drug molecules.

The hydrophobic DESs (L5, L7, and D4), characterized by weaker hydrogen bonding, exhibit notably low surface tension values (around 31 mN/m). This reduced surface tension indicates that less energy is required to expand the surface area of these liquids. This is advantageous for improving the wettability of poorly soluble drugs, ultimately enhancing their solubility and bioavailability.

The superior solubilizing effect of the D3 DESs on IBU stems from a synergistic combination of its low surface tension (30–31 mN/m) and distinct molecular interactions. The low surface tension facilitates better wetting and penetration of the drug particles. Simultaneously, the DESss components engage in favorable interactions with the IBU. The hydrophobic MNT interacts with the hydrophobic regions of the drug molecule, while the hydrophilic, long-chained PEG 400 acts as a flexible medium, accommodating IBU through various solubilization mechanisms, including hydrogen bonding and van der Waals forces.

These findings align with existing literature, which highlights the importance of low surface tension in DESss systems for enhancing the solubility of poorly soluble drugs [69,202].

Overall, the low surface tension in DESs systems is a pivotal factor that enhances the solubility of poorly soluble drugs, thereby improving their bioavailability and therapeutic efficacy, addressing one of the most significant challenges in pharmaceutical development [62].

#### 2.4.3. Thermal Analysis

The thermal behavior of the DESs systems under investigation was characterized using modulated temperature differential scanning calorimetry (MTDCS). This pivotal analytical technique provides insights into the thermal properties of DESs, including melting points (Tm), glass transition temperatures (Tg), and other phase transitions, which are crucial for understanding their overall properties. By measuring the heat flow associated with these transitions as a function of temperature, MTDSC can reveal critical characteristics such as melting point depression and the formation of eutectic systems, both of which signify strong intermolecular interactions within the NADESs–drug mixtures.

Table 4 provides a detailed thermal analysis of the DESs systems and their raw components during both the first heating and cooling cycles, including key parameters such as onset temperature (T_on_), melting temperature (T_m_) for heating, crystallization temperature (T_c_) for cooling, glass transition temperature (Tg), and the corresponding enthalpy (ΔH) and heat capacity changes (ΔCp). These values offer crucial insights into the thermal behavior, stability, and phase transitions of the DESs formulations, which are important for understanding their potential use in pharmaceutical applications.

**Figure 5:**
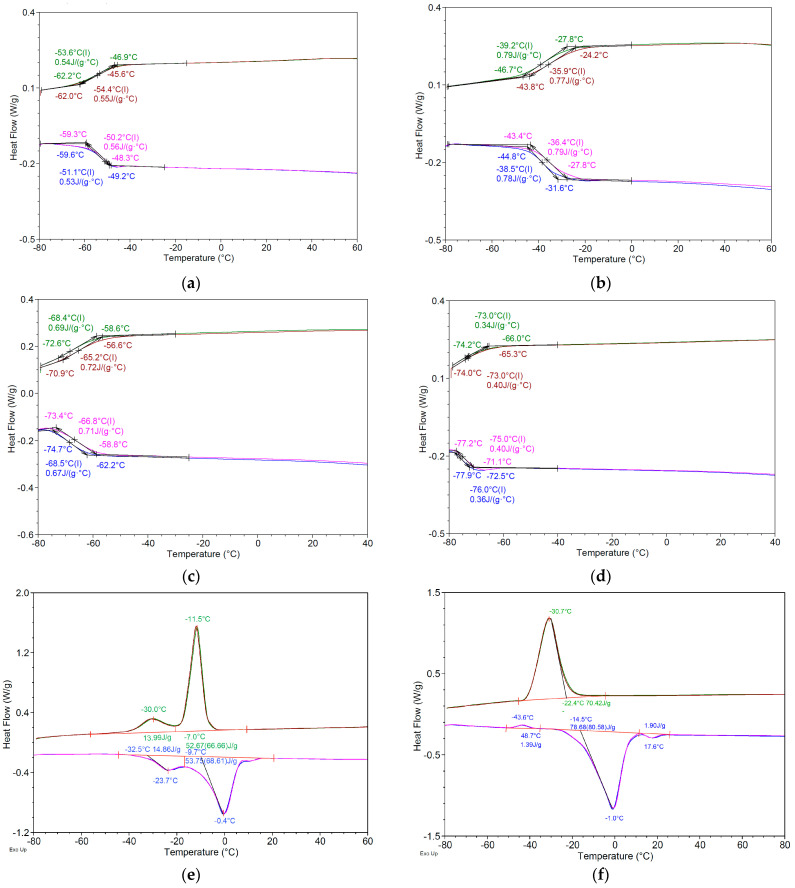
DSC thermograms of various DESs systems, including (**a**) A3—ChCl:oxalic acid (1:1), (**b**) A5—arginine:glycolic acid (1:8), (**c**) G3—ChCl:Sor (1:1), (**d**) G5—ChCl:Sor:Gly (2:1:1), (**e**) L5—MNT:Ola (1:2); (**f**) D3—MNT:PEG 400 (1:1). The thermograms are overlays of two heating–cooling cycles (green—first cooling cycle; brown—second cooling cycle; blue—first heating cycle; and pink—second heating cycle).

MTDSC analysis revealed that the hydrophilic NADESss systems exhibited a glass transition temperature rather than a distinct melting point, i.e., a temperature at which an amorphous material transitions from a hard and brittle state to a more pliable and rubber-like state [203]. This is a second-order transition, characterized by the absence of latent heat, and manifests as a step-like transition in the DSC thermogram (Table 4, Figure 5a–d).

This behavior is characteristic of NADESs, which are known to form highly viscous, supercooled liquids due to the strong intermolecular interactions, particularly hydrogen bonding, between their constituents [204]. Instead of a sharp transition from solid to liquid, NADESs undergo a gradual glass–rubbery transition over a temperature range defined by the Tg. Below the Tg, molecular mobility is restricted, resembling a solid-like state. As the temperature rises above the Tg, the NADESs progressively transitions to a more fluid, rubbery state with increased molecular mobility [205]. This unique characteristic of NADESs is crucial in determining the practical usability and stability of the systems, particularly in drug delivery, where the glassy or rubbery state can be leveraged to enhance drug solubility, control drug release, and improve formulation stability.

The MNT-based DESs systems exhibited distinct thermal behaviors, characterized by well-defined melting and crystallization temperatures (Table 4). The formation of eutectic mixtures was evidenced by a significant depression of melting points for all systems compared to their individual components. Furthermore, the presence of multiple crystallization and melting peaks in some systems, such as L5 and D3, indicated the coexistence of distinct phases, highlighting the multicomponent nature of these systems (Table 4, Figure 5e–f).

Focusing on the L5 system, two distinct endothermic events were observed during heating. The peak at −23.6 ± 0.4 °C, with a relatively low enthalpy change (ΔH = 14.84 ± 0.12 J/g), likely corresponds to the solid–solid phase transition of the γ polymorph of Ola to its α form, consistent with previous observations in similar systems [206]. The subsequent peak at −0.4 ± 0.2 °C, characterized by a significantly higher ΔH (53.70 ± 0.91 J/g), represents the melting of the primary eutectic phase formed by menthol and oleic acid. These observations underscore the complex molecular interactions within these DESs systems, particularly in the formation and melting of eutectic phases, which distinguish them from simple mixtures of their individual components.

### 2.5. Impact of Water on Physicochemical Properties of Hydrophilic NADESs

The incorporation of water into hydrophilic DESs, exerts a profound influence on their physicochemical properties [71,207]. Water acts as a plasticizer, disrupting the hydrogen bonding network within the DESs and effectively reducing viscosity, enhancing molecular mobility and impacting drug solubility [70]. Understanding these effects is crucial for tailoring DESs for specific applications, such as drug delivery, where solubility and the mass transfer of active pharmaceutical ingredients are paramount.

To investigate the influence of water on both drug solubility and the physicochemical properties of the eutectic systems, the G3 system, comprising ChCl and Sor in a 1:1 molar ratio was used as model. This sugar-based system exhibits strong hydrogen bonding and tight molecular packing, resulting in high viscosity, making it a suitable candidate to study the effects of water incorporation.

To create the ChCl:Sor:water mixtures, water was added dropwise to the G3 system, under continuous stirring, to achieve weight fractions of 5%, 10%, 15%, 20%, and 25% in the final system. We then assessed the impact of water on the viscosity and thermal behavior of the resulting systems, as well as on the solubility of IBU and MFA.

**Figure 6:**
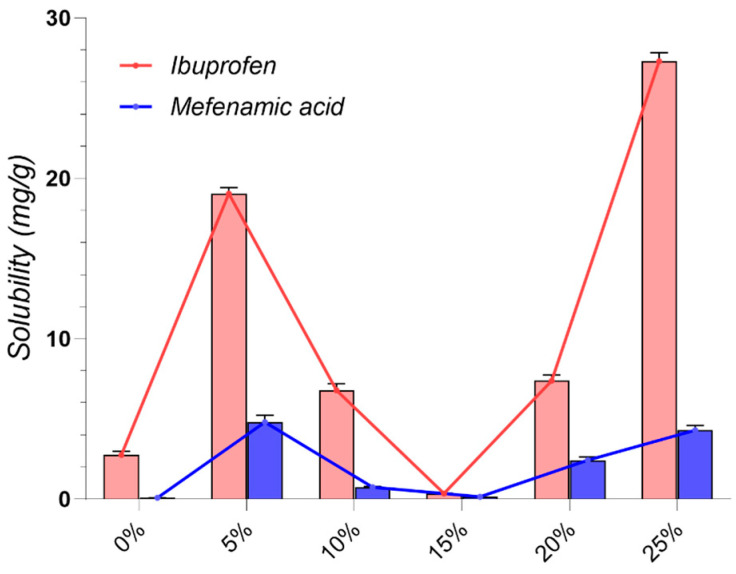
Influence of added water content (expressed as weight ratio) on the solubility of IBU and MFA in ChCl:Sor (1:1) NaDESs.

Our findings, illustrated in Figure 6 underscore the significant impact of water content on drug solubility. In the absence of water, both drugs exhibit limited solubility (2.76 ± 0.005 and 0.082 ± 0.002 mg/g, respectively). However, the introduction of just 5% water dramatically enhances the solubility of both drugs. IBU solubility experiences a nearly seven-fold increase, while MFA solubility sees an impressive sixty-fold surge. This remarkable enhancement stems from the disruption of the tightly packed hydrogen bonding network between ChCl and Sor. In the binary mixture, strong intermolecular interactions between the components form a dense and rigid hydrogen bond network [176]. This dense packing restricts free volume, limiting the available space for drug molecules to dissolve [192]. Water molecules, acting as a plasticizer, interrupt these strong hydrogen bonds, thereby “loosening” the network and creating additional free volume within the eutectic system, providing additional space for drug molecules to fit into the solvent matrix [208], thereby improving solubility.

Interestingly, a further increase in water content to 10% and 15% results in a decrease in drug solubility (Figure 6). This observation suggests that at these levels, water may begin to disrupt the eutectic structure, leading to less effective solvation. However, as water content rises further to 20% and 25%, drug solubility increases again, peaking at 25%. This complex trend highlights the intricate relationship between DESs structure and water content. While moderate amounts of water enhance solubility, excessive water can disrupt the delicate balance of interactions within the DES. The subsequent solubility increase at higher water concentrations may be attributed to a shift in the solvation mechanism or the formation of new favorable interactions [71].

**Figure 7:**
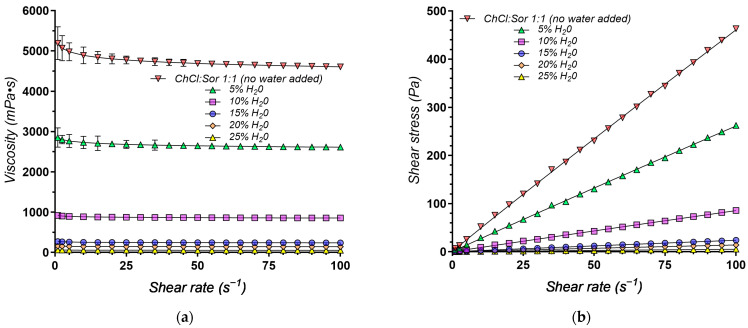
Rheological properties of the ChCl:Sor (1:1) eutectic system at various water concentrations (37 °C) represented as (**a**) viscosity (mPa·s) vs. shear rate (s^−1^) and (**b**) shear stress (Pa) vs. shear rate (s^−1^).

Given that water content is a key determinant of DESs viscosity, a crucial parameter influencing its pharmaceutical applicability, we further investigated this relationship. Figure 7 illustrates the impact of increasing water content up to 25% on the viscosity of the ChCl:Sor (1:1) NADESs. The data reveal a significant influence of water content on the viscosity and shear stress behavior of the eutectic mixture.

As expected, the anhydrous system exhibits the highest viscosity (Figure 7). However, the addition of water causes a substantial decrease in viscosity. This decrease is most pronounced at lower water concentrations, indicating that even small additions of water can significantly impact the system’s rheological behavior. This trend is further corroborated by analyzing the rheological parameters, specifically the consistency index (K), obtained from the Power-law model (Table 5). As the water content increases from 0% to 25%, there is a dramatic decrease in K, a direct measure of viscosity, from 5.191 Pa·s^n^ (no water) to 0.061 Pa·s^n^ (25% water).

Furthermore, the flow behavior index (n), ranging between 0.9687 and 0.9846, suggests Newtonian flow characteristics. This implies that the viscosity remains constant regardless of the applied shear rate (Figure 7).

This decrease in viscosity, brought about by the addition of water, is particularly beneficial for applications requiring fluidity and ease of handling. For instance, in drug delivery systems, lower viscosity can facilitate higher drug loading and more efficient release. The observed decrease in shear stress at higher water concentrations further supports this, highlighting the role of water in modulating the physical properties of DESs and making them suitable for specific applications.

MTDSC was employed to investigate the thermal properties of G3 eutectic mixtures in the presence of varying water content (0%, 5%, 10%, 15%, 20%, and 25%). The analysis focused on observing the phase transitions during heating and cooling cycles, providing insights into the impact of water on the eutectic system’s thermal behavior.

**Figure 8:**
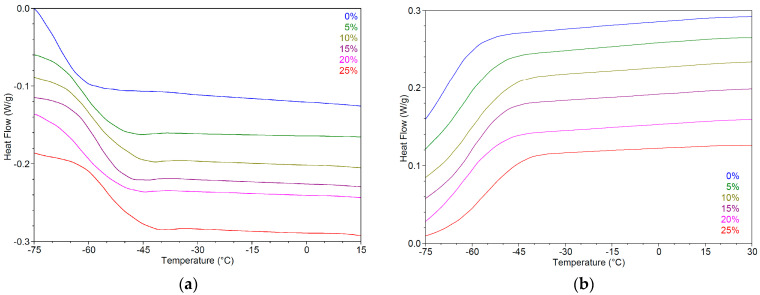
DSC thermograms displaying the thermal properties of ChCl:Sor (1:1) eutectic mixtures with varying water content (0%, 5%, 10%, 15%, 20%, and 25%); (**a**) first heating cycle showing endothermic shifts corresponding to the glass transitions of the eutectic mixtures; (**b**) first cooling cycle depicting exothermic shifts representing the glass transitions during cooling.

Figure 8 displays the DSC thermograms for the first heating and first cooling cycles, respectively. Each curve represents a different water content, indicating significant changes in the thermal behavior with increasing water concentration.

During the first heating cycle (Figure 8a), all samples exhibit endothermic shifts corresponding to the glass transitions of the eutectic mixtures. In the first cooling cycle (Figure 8b), the exothermic shifts are observed, representing the glass transitions during cooling.

The data reveal an apparent trend of increasing glass transition temperatures (Tg) with higher water content across both heating and cooling cycles. This trend is summarized in Table 6, which presents the onset temperature (Ton), midpoint temperature (Tg), and the change in heat capacity (ΔCp) for both heating and cooling cycles.

Contrary to the typical effect where the addition of a plasticizer lowers the Tg [205], our results reveal an apparent nonlinear trend of Tg shifting toward higher temperatures with increasing water content (G3-0 vs. G3-25, *p* < 0.0001).

The introduction of water could be inducing a rearrangement of ChCl and Sor molecules, resulting in a more tightly packed structure with stronger intermolecular interactions and a higher Tg. This can be attributed to the antiplasticizer effect of water and Sor interactions [209,210], enhancing the thermal stability of the eutectic mixtures. The findings highlight the complex nature of plasticization effects within NADESs, as well as the importance of considering water content and its interactions with eutectic components in the design of DESs-based pharmaceutical formulations. By tuning the water content, it is possible to optimize both the solubility of active pharmaceutical ingredients and the rheological properties of the solvent system. This tunability makes DESs attractive for developing customized drug delivery systems with specific release profiles and handling characteristics.

## 3. Materials and Methods

### 3.1. Materials

Ibuprofen (IBU) powder ≥98% (Sigma-Aldrich, Saint Louis, MO, USA) and mefenamic acid (MFA) 98% (MP Biomedicals, Eschwege, Germany) were utilized as model drugs in the study.

Various HBA and HBD compounds for DESs preparation were sourced from different suppliers as follows: choline chloride ≥ 98%, glycolic acid p.a., glycerol ≥ 99%, lactic acid > 85%, L-cysteine, oxalic acid, and xylitol were purchased from Sigma-Aldrich (Saint Louis, MO, USA); high-purity D-(−)-Sor ≥ 96%, D-(−)-fructose, and D-(+)-glucose from VWR Chemicals (Solon, OH, USA); isopropyl myristate (IPM) and (R)-(+)-limonene from Merck (Darmstadt, Germany); DL-menthol >98% from Thermo Fisher Scientific (Waltham, MA, USA); PEG 400 and propylene glycol (PG) from Scharlau (Sentmenat, Spain); L-arginine from MP Biomedicals (Eschwege, Germany); decanoic acid from Alfa Aesar (Kandel, Germany); and Miglyol^®^ 812 (MCT) from IOI Oleo GmbH (Hamburg, Germany).

For the HPLC analysis, acetonitrile and methanol (HPLC gradient grade purity) were sourced from Honeywell Research Chemicals (Seelze, Germany), while formic acid (LC-MS grade) was obtained from Fisher Chemical (Pardubice, Czech Republic). Ultrapure water (18.2 MΩ·cm at 25 °C, TOC < 5 ppb) was produced using a Milli-Q EQ 7008 purification system (Merck Millipore, Burlington, MA, USA).

### 3.2. Preparation of DESs

Our study primarily focused on evaluating the solubilizing potential of both hydrophilic and hydrophobic NADESs, as more biocompatible alternatives for pharmaceutical use [36]. We prepared a range of NADES systems, including organic acid-based (A1–A7), sugar- and sugar alcohol-based (G1–G12), and hydrophobic MNT-based (L1–L11) formulations. Additionally, given their widespread use in the pharmaceutical industry, we included several synthetic alcohols and esters, specifically PEG 400, PG, and IPM in our analysis (D1–D4). The composition of the specific evaluated DESs is presented in Table 1.

The DES systems were prepared using a modified heating method [17]. Briefly, accurately weighed amounts of each component, as required to achieve the molar ratios indicated in Table 1, were combined in 40 mL screw-cap glass vials and heated to 75 °C under constant stirring at 1000 rpm using a ThermoMixer C system (Eppendorf, Hamburg, Germany) system until a clear, homogeneous liquid was formed, with synthesis times varying from approximately 30 min for hydrophobic NADESs and the synthetic DESs systems to 2 h for organic acid-based NADESs and up to 12 h for sugar- and sugar alcohol-based NADESs.

Prior to use, ChCl was dried under vacuum at 50 °C for a minimum of 24 h and stored in an airtight container. Sugars were finely ground using a mortar and pestle to increase surface area and facilitate eutectic formation. All other chemicals were used as received without further purification. Prepared DESs were stored in airtight vials within a desiccator until further use.

### 3.3. Equilibrium Solubility Studies

The equilibrium solubility of both IBU and MFA in each DES was determined using a shake-flask method. An excess amount of drug was added to 750 mg of each DES in a 2 mL Eppendorf tube. The mixtures were vortexed for 15 min at 2500 rpm to ensure thorough mixing. Following vortexing, the samples were maintained for 24 h at 25 °C and 1000 rpm in a thermal mixer to reach equilibrium solubility. The samples were further centrifuged at 15,000 rpm for 10 min to separate undissolved drug. Approximately 50 mg of the supernatant was carefully transferred to a 5 mL volumetric flask and diluted to volume with an appropriate solvent, depending on the DESs composition and drug solubility. The resulting solutions were further diluted as needed to fall within the linear range of the preestablished calibration curves, and filtered using a 0.22 μm polyethersulfone syringe filter. Finally, 5 µL of each diluted sample was injected into the HPLC system for analysis.

### 3.4. Drug Quantification

The quantification of the amount of drug solubilized in the eutectic mixtures was performed by HPLC. This approach was necessary due to the significant UV absorbance of most eutectic mixture components, which would interfere with direct UV spectrophotometric analysis. The HPLC methods for both IBU and MFA were validated according to the current ICH guidelines [211] to ensure linearity, accuracy, precision, selectivity, and sensitivity.

A Jasco 4000 Series RP-HPLC system (JASCO Corporation, Tokyo, Japan) equipped with a quaternary pump (model PU-4180), a thermostated autosampler (AS-4150), a column oven (CO-4061), and a diode array detector (MD-4010) was used for the analysis.

Chromatographic separation was achieved on a 100 × 3 mm Kinetex^®^ C18 column with a particle size of 2.6 µm (Phenomenex, Torrance, CA, USA) maintained at 45 °C. The mobile phase consisted of 0.1% formic acid (mobile phase A) and a 1:1 (*v*/*v*) mixture of acetonitrile and methanol (mobile phase B). A gradient elution program was employed, starting from a mobile phase composition of 45:55 (A:B, *v*/*v*) and ending at 95% mobile phase B to ensure the complete removal of any lipid components present in the eutectic mixtures. Detection wavelengths were set at 222 nm for IBU and 280 nm for MF.

The quantification of MFA and IBU dissolved in each DES system was performed by interpolating peak areas against calibration curves constructed using standard solutions within the concentration range of 0.5–100 µg/mL for MFA and 1–100 µg/mL for IBU. The results were expressed as mg drug per gram of drug-saturated DES. All analyses were performed in triplicate. The accuracy and precision (evaluated on two levels: repeatability and intermediate precision) were evaluated using quality control (QC) samples at low (LQC), medium (MQC), and high (HQC) concentration levels. Five replicates for each QC sample were used for the analysis. The mean percent recovery of the analyte in the QC samples was used to evaluate the accuracy of the method, while precision was estimated in terms of relative standard deviation (RSD, %). The main results for the HPLC validation methods for IBU and MFA are presented in the Supplementary Material.

### 3.5. Measurement of DESs Density

The density of each DES was determined gravimetrically. A known volume of DESs was precisely transferred into a pre-weighed glass beaker using a Repeater^®^ M4 Multi-Dispenser Pipette (Eppendorf, Hamburg, Germany) equipped with ViscoTip^®^ dispensing tips, specifically designed and optimized for the accurate handling of highly viscous liquids. The mass of the dispensed DESs was measured using an XA 210.5Y.A analytical balance (Radwag, Radom, Poland).

### 3.6. Rheological Evaluation

The viscosity and flow behavior of selected representative DESs systems was tested using an RM100 Plus viscometer, equipped with the MS DIN 19 measuring system (Lamy Rheology Instruments, Champagne au Mont d’Or, France). Flow curves were obtained by applying a shear rate ramp in the range 1–100 s^−1^. A CT-DIN water jacket (Lamy Rheology Instruments, Champagne au Mont d’Or, France) connected to a Corio CD recirculating water bath (Julabo, Seelbach, Germany) ensured a constant temperature throughout the measurements.

The recorded rheological data, including shear rate (s^−1^), shear stress (Pa), and viscosity (Pa·s), were recorded and analyzed using the Power law model [212] to describe the flow behavior of the DESs (Equation (1)):

(1) $$\tau =K\cdot {\dot{\gamma }}^{n}$$

where τ (Pa) is the shear stress, representing the force per unit area applied to the fluid, $\dot{\gamma }$ is the shear rate (s^−1^), K is the consistency index (Pa·s^n^), which reflects the viscosity of the fluid at a shear rate of 1 s^−1^, and n represents the flow behavior index (dimensionless).

For n = 1, the equation reduces to $\tau =K\cdot \dot{\gamma }$, which implies that viscosity is constant and independent of the shear rate, which is characteristic of Newtonian fluids, while n < 1 indicates a shear-thinning (pseudoplastic) behavior. The degree of pseudoplasticity increases as the value of ‘n’ deviates further from unity [213].

### 3.7. Surface Properties

The surface properties of the DESs, specifically their surface tension and contact angle, were determined using a CAM-101 Goniometer equipped with a Hamilton syringe, a C209-30 needle, and a digital camera (KSV Instruments Ltd., Espoo, Finland), as previously reported [214,215].

The pendant drop method was employed to measure the surface tension of the DESs [212,216]. Briefly, a small droplet of each DESs was suspended from the tip of the syringe needle, and the digital camera captured an image of the droplet profile. The integrated curve-fitting software then analyzed the droplet shape using the Young–Laplace equation (Equation (2)) to calculate the surface tension (ST):

(2) $${∆}_{p}=p_{\mathrm{int}}-p_{\mathrm{ext}}=\gamma_{\mathrm{LG}}\left(\frac{1}{r_{1}}+\frac{1}{r_{2}}\right)$$

where Δp represents the Laplace pressure as the pressure difference between internal and external areas of a curved liquid, γLG represents the surface tension at the liquid/gas interface, and r_1_, r_2_ represent the principal radii of curvature.

The results were reported in triplicate at the operating temperature of 25 ± 0.5 °C.

### 3.8. Thermal Analysis

To assess the thermal behavior of the NADESss, modulated temperature differential scanning calorimetry (MTDSC, ±0.796 °C/30 s) experiments were carried out with a DSC Q2000 system equipped with RCS 90 cooling system (TA Instruments, New Castle, DE, USA). Samples (10–15 mg) were sealed in Tzero aluminum pans with hermetic lids. A heat–cool–heat (HCH) approach was used in a single run, as follows: the samples were cooled to −80 °C, equilibrated for 3 min, heated to between 70 °C and 280 °C (depending on the analyzed sample) at a heating rate of 10 °C/min (first heating), equilibrated for 3 min, cooled to −80 °C at 10 °C/min (cooling cycle), equilibrated for 3 min, and heated again at 10 °C/min (second heating). A helium 5.0 purge gas (99.999% purity) was maintained at a flow rate of 25 mL/min throughout the analysis.

### 3.9. Statistical Analysis

Data visualization and statistical analysis were performed using GraphPad Prism 9.3.1 (GraphPad Software, San Diego, CA, USA). Statistical significance was determined using a one-way analysis of variance with a post-hoc Tukey test with a significance level of *p* < 0.05.

## 4. Conclusions

This study has successfully demonstrated the potential of various pharmaceutically acceptable DESs systems to significantly enhance the solubility of IBU and MFA., thus addressing a critical challenge in improving their bioavailability and therapeutic efficacy.

Our findings reveal that hydrophobic DESs systems exhibited the most substantial solubility enhancements. Notably, IBU solubility reached 379.69 ± 22.718 mg/g in an MNT: PEG 400 (1:1) mixture (D3), representing a greater than 6700-fold increase compared to its aqueous solubility. Similarly, MFA solubility in the MNT: MCT (1:1) system (L7) demonstrated a remarkable 400-fold enhancement.

Furthermore, we demonstrate the critical role of water as a modifier in sugar-based NADESs systems, where controlled water addition can modulate both drug solubility and the system’s viscosity. A seven-fold increase in IBU solubility and a sixty-fold increase in MFA solubility were achieved at 5% water concentrations as compared to anhydrous systems; viscosity decreased abruptly with increased water content. This tunability makes DESs highly versatile for various drug delivery applications, ranging from immediate-release to controlled-release formulations.

Beyond their remarkable impact on solubility, our study also revealed the tunable rheological properties of DESs, making them highly versatile for various drug delivery applications. Hydrophobic DESs systems, characterized by their near-Newtonian flow behavior and low viscosities (<100 mPa·s), are particularly well-suited for immediate-release formulations, as they promote rapid drug diffusion. Conversely, systems with higher viscosities, such as those based on sugars, sugar alcohols, and Arg, hold significant promise for controlled-release formulations.

Importantly, this study extends beyond solubility enhancement, underscoring the multifaceted advantages of DESs in drug delivery. Their intrinsic antimicrobial activity, coupled with their potential as permeation enhancers and taste-masking agents, further strengthens their position as promising candidates for future drug delivery systems.

In conclusion, our findings provide compelling evidence for the transformative potential of DESs in addressing the persistent challenge of poorly soluble drugs. A further exploration of novel DESs combinations, their incorporation into specific drug delivery systems, and comprehensive in vitro and in vivo studies are warranted to fully realize their therapeutic potential and ultimately improve patient care by enhancing the delivery and efficacy of poorly soluble drugs.

## Figures and Tables

**Table 3:** Rheological behavior of various DESs systems modeled using the Power-law model. The table includes the consistency index (K), flow behavior index (n), and the coefficient of determination (R^2^) for each evaluated DESs composition.

Sample	Components				K (Pa·s^n^)	n	R^2^
	1	2	3	Molar Ratio			
A1	ChCl	Gla	-	1:1	0.154	0.9216	0.9994
A2	ChCl	Gla	-	1:2	0.196	0.9159	0.9985
A3	ChCl	Oxa		1:1	0.172	0.9566	0.9971
A5	Arg	Gla		1:8	0.949	0.9743	0.9994
G1	ChCl	Gly		1:2	0.221	0.9200	0.9983
G3	ChCl	Sor		1:1	5.191	0.9741	0.9993
G5	ChCl	Glu	Gly	2:1:1	0.951	0.9846	0.9997
G9	ChCl	Sor	Water	1:1:1	1.901	0.9810	0.9982
L5	MNT	Ola	-	1:2	0.072	0.8513	0.9968
L7	MNT	MCT	-	1:1	0.058	0.8651	0.9974
D3	MNT	PEG 400	-	1:1	0.126	0.7603	0.9989
D4	MNT	IPM	-	1:1	0.031	0.9380	0.9976

**Table 4:** Thermal analysis of various DESs systems and raw components during the first heating and cooling cycles.

	First Heating						First Cooling					
	T_on_ (°C)	T_m_ (°C)	ΔH J/g	T_on_ (°C)	T_g_ (°C)	ΔC J/(g·°C)	T_on_ (°C)	T_c_ (°C)	ΔH J/g	T_on_ (°C)	T_g_ (°C)	ΔC J/(g·°C)
A1	-	-	-	−78.5 ± 0.8	−70.7 ± 0.6	0.48 ± 0.02	-	-	-	−76.4 ± 0.6	−67.1 ± 0.6	0.51 ± 0.02
A2	-	-	-	−71.8 ± 0.5	−65.7 ± 0.4	0.51 ± 0.01	-	-	-	−73.9 ± 0.4	−65.7 ± 0.4	0.55 ± 0.03
A3	-	-	-	−59.7 ± 0.8	−51.0 ± 0.5	0.53 ± 0.02	-	-	-	−46.8 ± 0.3	−53.5 ± 0.3	0.53 ± 0.02
A5	-	-	-	−44.6 ± 0.5	−38.3 ± 0.1	0.76 ± 0.03	-	-	-	−27.6 ± 0.5	−39.1 ± 0.5	0.78 ± 0.03
G1	-	-	-	-	-	-	-	-	-	-	-	-
G3	-	-	-	−74.5 ± 0.7	−68.3 ± 0.5	0.65 ± 0.03				−58.4 ± 0.5	−68.3 ± 0.3	0.68 ± 0.03
G5	-	-	-	−77.7 ± 1.0	−76.1 ± 0.3	0.37 ± 0.02	-	-	-	−66.2 ± 0.8	−73.2 ± 0.5	0.34 ± 0.01
G9	-	-	-	−70.6 ± 0.9	−62.3 ± 0.4	0.58 ± 0.02				−54.7 ± 0.9	−63.7 ± 0.6	0.59 ± 0.02
L5	−32.7 ± 1.1	−23.6 ± 0.4	14.84 ± 0.12				−7.0 ± 0.3	−11.5 ± 0.2	52.60 ± 1.02			
	−9.7 ± 0.4	−0.4 ± 0.2	53.70 ± 0.91				−22.1 ± 0.3	−30.0 ± 0.1	13.90 ± 0.34			
L7	−17.8 ± 0.4	−10.3 ± 0.3	45.13 ± 0.65				−42.0 ± 0.3	−48.8 ± 0.3	27.99 ± 0.92			
D3	−14.7 ± 0.7	−1.1 ± 0.4	78.74 ± 1.02	-	-	-	−22.2 ± 0.7	−30.7 ± 0.3	70.42 ± 1.26	-	-	-
	12.7 ± 0.5	17.6 ± 0.3	1.90 ± 0.04	-	-	-	-	-	-	-	-	-
D4	−6.5 ± 0.3	−0.9 ± 0.2	113.40 ± 3.28				−6.1 ± 0.3	−16.7 ± 0.2	136.97 ± 4.25			
ChCl	63.4 ± 0.8	68.7 ± 0.8	112.41 ± 2.93	-	-	-	-	-	-	-	-	-
	179.0 ± 0.5	182.8 ± 0.5	103.71 ± 3.12	-	-	-	-	-	-	-	-	-
MNT	31.2 ± 0.4	36.1 ± 0.3	87.33 ± 1.56	-	-	-	20.7 ± 0.4	17.2 ± 0.3	62.96 ± 1.67	-	-	-
Gla	-	-	-							-	-	-
Oxa	186.0 ± 0.3	191.2 ± 0.5	69.44 ± 1.3	-	-	-	-	-	-	-	-	-
Arg	212.0 ± 0.5	221.3 ± 0.7 *	-	-	-	-	-	-	-	-	-	-
Gly	-	-	-	−84.4 ± 0.8	−82.7 ± 0.6	0.37 ± 0.01	-	-	-	-	-	-
Sor	94.4 ± 0.2	99.2 ± 0.2	169.33 ± 0.32	-	-	-	-	-	-	1.3 ± 0.8	−2.6 ± 0.7	1.02 ± 0.02
Ola	−25.6 ± 0.5	−18.5 ± 0.3	12.41 ± 0.41	-	-	-	0.5 ± 0.2	−2.8 ± 0.3	91.43 ± 1.42	-	-	-
	−0.1 ± 0.3	6.4 ± 0.2	88.80 ± 0.71	-	-	-	−17.3 ± 0.3	−23.0 ± 0.2	11.63 ± 0.32			
MCT	−11.0 ± 0.5	−1.9 ± 0.3	76.07 ± 0.94	-	-	-	−37.8 ± 0.2	−42.1 ± 0.3	54.46 ± 0.81	-	-	-
PEG 400	−12.4 ± 0.6	5.0 ± 0.5	92.68 ± 1.78	-	-	-	−13.7 ± 0.5	−20.4 ± 0.5	82.94 ± 1.59	-	-	-

* decomposition

**Table 5:** Rheological behavior of ChCl:Sor (1:1) eutectic system at various water concentrations modeled using the Power-law model. The table includes the consistency index (K), flow behavior index (n), and the coefficient of determination (R^2^) for each evaluated DESs composition.

Code	Components			K (Pa·s^n^)	n	R^2^
	1	2	Weight Ratio			
G3-0	G3	-	-	5.191	0.9741	0.9993
G3-5	G3	water	95:5	2.851	0.9810	0.9991
G3-10	G3	water	90:10	0.917	0.9846	0.9989
G3-15	G3	water	85:15	0.270	0.9727	0.9978
G3-20	G3	water	80:20	0.158	0.9748	0.9993
G3-25	G3	water	75:25	0.061	0.9687	0.9984

**Table 6:** Glass transition temperatures (Tg) and changes in heat capacity (ΔCp) of G3 eutectic mixture with varying water content (0%, 5%, 10%, 15%, 20%, and 25%), during the first heating and cooling cycles.

Sample Code	First Heating			First Cooling		
	T_on_ (°C)	T_g_ (°C)	ΔC J/(g·°C)	T_on_ (°C)	T_g_ (°C)	ΔC J/(g·°C)
G3-0	−74.5 ± 0.7	−68.3 ± 0.5	0.65 ± 0.03	−58.4 ± 0.5	−68.3 ± 0.3	0.68 ± 0.03
G3-5	−68.4 ± 0.6	−60.6 ± 0.4	0.59 ± 0.02	−52.2 ± 0.4	−61.4 ± 0.2	0.60 ± 0.02
G3-10	−67.6 ± 0.6	−59.9 ± 0.2	0.64 ± 0.02	−49.2 ± 0.5	−62.1 ± 0.3	0.70 ± 0.02
G3-15	−65.4 ± 0.5	−57.2 ± 0.2	0.64 ± 0.02	−50.5 ± 0.5	−60.5 ± 0.3	0.63 ± 0.01
G3-20	−70.0 ± 0.4	−62.7 ± 0.3	0.61 ± 0.01	−51.3 ± 0.4	−59.8 ± 0.2	0.62 ± 0.02
G3-25	−63.5 ± 0.7	−55.1 ± 0.5	0.61 ± 0.02	−44.6 ± 0.4	−58.1 ± 0.2	0.59 ± 0.02

## Data Availability

The original contributions presented in the study are included in the article; further inquiries can be directed to the corresponding author.

## Supplementary Materials

The following supporting information can be downloaded at: https://www.mdpi.com/article/10.3390/ph17101316/s1, Figure S1: Calibration curve for the HPLC quantification of IBU; Figure S2: Representative overlayed chromatograms of IBU standard samples versus sample matrix blank; Figure S3: Calibration curve for the HPLC quantification of MFA; Figure S4: Representative overlayed chromatograms of MFA standard samples versus sample matrix blank; Table S1: Regression statistics for the IBU calibration curve; Table S2: Evaluation of the accuracy and precision of the IBU HPLC method; Table S3: Regression statistics for the MFA calibration curve; Table S4: Evaluation of the accuracy and precision of the MFA HPLC method.
