# Supplementary material for: Exploring Deep Eutectic Solvents as Pharmaceutical Excipients: Enhancing the Solubility of Ibuprofen and Mefenamic Acid
Source: Pharmaceuticals (Basel). 2024 Oct 2;17(10):1316. doi: 10.3390/ph17101316 (PMC11510164; doi:10.3390/ph17101316)
Supplement: Supplementary file 1 [file pharmaceuticals-17-01316-s001.zip › pharmaceuticals-3155877-supplementary.pdf]

## A. Ibuprofen (IBU) HPLC method validation:

### Linearity

The calibration curve for ibuprofen (IBU) quantification was constructed by analyzing seven standard solutions in triplicate, covering a concentration range of 1-100 µg/mL (1, 2.5, 5, 10, 25, 50 and 100 µg/mL respectively). The linearity of the method was confirmed by the high correlation coefficient ($R^2$ = 0.9996), indicating excellent linearity across the working range. The equation of the calibration curve was Y = 13628.52 X + 379.62. The regression analysis of the calibration curve experimental results is presented in Table S1.

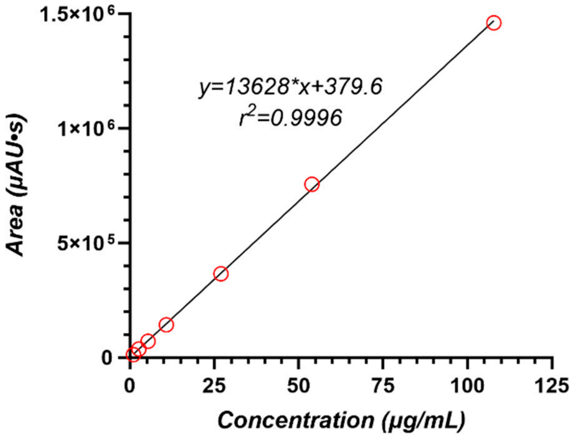


**Figure S1.** Calibration curve for the HPLC quantification of IBU

**Table S1.** Regression statistics for the IBU calibration curve

| *Regression Statistics* | |
|---|---|
| Multiple R | 0.9997 |
| R Square | 0.9996 |
| Adjusted R Square | 0.9996 |
| Standard Error | 3158.60 |
| Observations | 7 |

| ANOVA | *df* | *SS* | *MS* | *F* | *Significance F* |
|---|---|---|---|---|---|
| Regression | 1 | 1.46887E+12 | 1.47E+12 | 147229.34 | 2.2818E-12 |
| Residual | 5 | 49883778.52 | 9976756 | | |
| Total | 6 | 1.46892E+12 | | | |

| | *Coefficients* | *Standard Error* | *t Stat* | *P-value* | *Lower 95%* | *Upper 95%* |
|---|---|---|---|---|---|---|
| Intercept | 379.62 | 1545.71 | 0.25 | 0.8158 | -3593.77 | 4353.00 |
| X Variable | 13628.52 | 35.52 | 383.70 | 0.0000 | 13537.22 | 13719.82 |

No endogenous signals interfered with the IBU chromatographic peaks, confirming the selectivity of the method. Overlayed chromatograms of IBU standards and a representative blank matrix sample are shown in Figure S2.

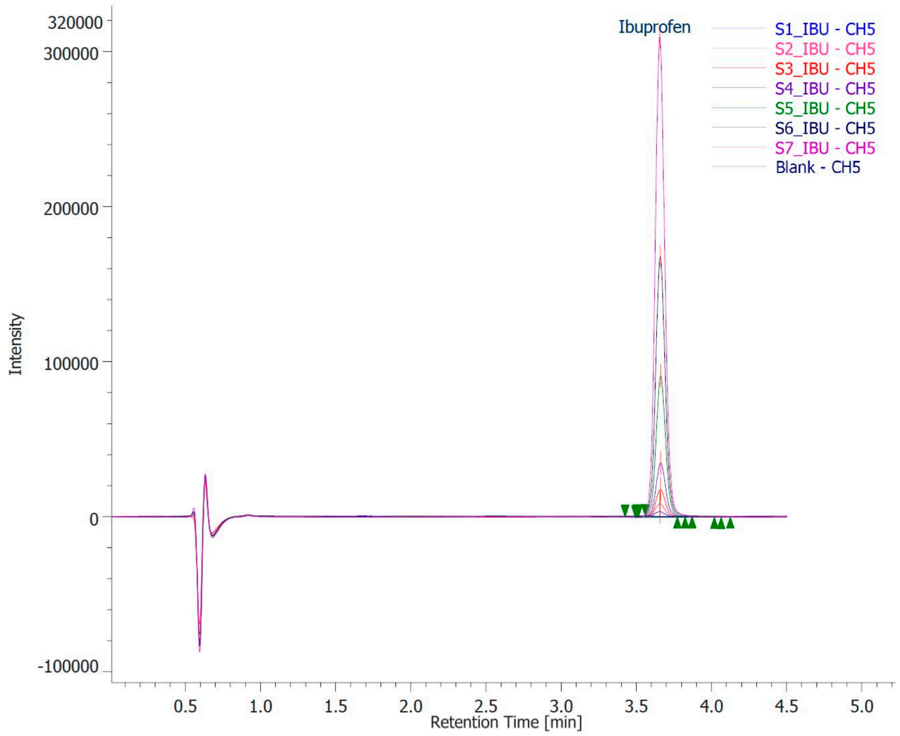


**Figure S2.** Representative overlayed chromatograms of IBU standard samples versus sample matrix blank

**Detection and Quantitation Limits:**

The values of the detection limit (DL) estimated based on considering a 3:1 Signal to Noise (S/N) ratio was 0.18 μg/mL while the quantitation limit (QL), evaluated at 10:1 S/N ratio was 0.47 μg/mL.

Based on the standard deviation (SD) of the linear response and slope of the calibration curve, values for the calculated DL and QL were somewhat bigger.

Detection limit (DL) was expressed as:

$$DL = \frac{3\sigma}{S}$$

and the quantitation limit (QL) was evaluated as:

$$QL = \frac{10\sigma}{S}$$

were

σ = the standard deviation of the response

S = the slope of the calibration curve

For IBU determination, estimated DL was 0.76 μg/mL, while QL was estimated as 2.32 μg/mL.

**Accuracy and Precision**

The accuracy and precision (evaluated on two levels: repeatability and intermediate precision) were evaluated using quality control (QC) samples at low (LQC, 9 μg/mL), medium (MQC, 45 μg/mL), and high (HQC, 75 μg/mL) concentration levels. Five replicates for each QC samples were used for the analysis. Mean percent recovery of the analyte in the QC samples was used to evaluate the accuracy of the method. Precision was estimated in terms of relative standard deviation (RSD, %). The experimental results are depicted in table S2.

**Table S2**. Evaluation of the accuracy and precision of the IBU HPLC method

| Sample level | Repeatability | | | | Intermediate precision | | | |
|---|---|---|---|---|---|---|---|---|
| | Calculated concentration (µg/mL) | Recovery (%) | Mean recovery (%) | % RSD | Calculated concentration (µg/mL) | Recovery (%) | Mean recovery (%) | % RSD |
| **LQC 9 µg/mL** | 9.02 | 100.20 | 100.58 | 0.54 | 8.86 | 98.44 | 99.17 | 1.11 |
| | 8.99 | 99.85 | | | 9.01 | 100.16 | | |
| | 9.07 | 100.77 | | | 9.05 | 100.56 | | |
| | 9.08 | 100.89 | | | 8.84 | 98.18 | | |
| | 9.11 | 101.19 | | | 8.87 | 98.51 | | |
| **MQC 45 µg/mL** | 45.08 | 100.18 | 99.80 | 0.31 | 44.75 | 99.44 | 99.53 | 0.59 |
| | 45.02 | 100.04 | | | 45.22 | 100.50 | | |
| | 44.86 | 99.70 | | | 44.79 | 99.54 | | |
| | 44.74 | 99.41 | | | 44.61 | 99.14 | | |
| | 44.85 | 99.67 | | | 44.56 | 99.02 | | |
| **HQC 75 µg/mL** | 74.85 | 99.81 | 99.61 | 0.12 | 75.05 | 100.07 | 99.32 | 0.74 |
| | 74.71 | 99.61 | | | 74.77 | 99.69 | | |
| | 74.69 | 99.59 | | | 74.41 | 99.21 | | |
| | 74.62 | 99.50 | | | 73.59 | 98.12 | | |
| | 74.65 | 99.53 | | | 74.61 | 99.49 | | |

The results demonstrate the high accuracy (with mean recoveries in the 99-101% range) and precision (RSD<1% for repeatability and <1.5% for intermediate precision) of the method for all QC levels.

## B. Mefenamic acid (MFA) HPLC method validation

### Linearity

The calibration curve for MFA quantification was constructed by analyzing eight standard solutions in triplicate, covering a concentration range of 0.5-100 µg/mL (0.5, 1, 2.5, 5, 10, 25, 50 and 100 µg/mL respectively). The linearity of the method was confirmed by the high correlation coefficient ($R^2 = 0.9995$), indicating excellent linearity across the working range. The equation of the calibration curve was Y = 37705 X -1258 (Figure S3). The regression analysis of the calibration curve experimental results is presented in Table S3.

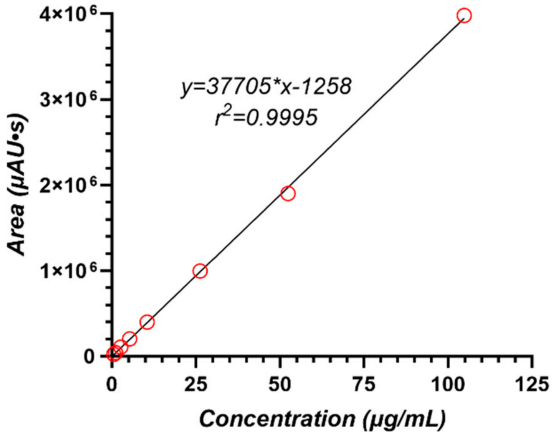


**Figure S3.** Calibration curve for the HPLC quantification of MFA

**Table S3.** Regression statistics for the MFA calibration curve

| *Regression Statistics* | |
|---|---|
| Multiple R | 0.9996 |
| R Square | 0.9995 |
| Adjusted R Square | 0.9995 |
| Standard Error | 17735.02 |
| Observations | 8 |

| *ANOVA* | *df* | *SS* | *MS* | *F* | *Significance F* |
|---|---|---|---|---|---|
| Regression | 1 | 1.22E+13 | 1.22E+13 | 38660.05 | 1.17E-12 |
| Residual | 6 | 1.89E+09 | 3.15E+08 | | |
| Total | 7 | 1.22E+13 | | | |

| | *Coefficients* | *Standard Error* | *t Stat* | *P-value* | *Lower 95%* | *Upper 95%* |
|---|---|---|---|---|---|---|
| Intercept | -1258.15 | 7806.53 | -0.16 | 0.8772 | -20360.05 | 17843.75 |
| X Variable | 37705.41 | 191.76 | 196.62 | 0.0000 | 37236.17 | 38174.64 |

No endogenous signals interfered with the MFA chromatographic peaks. Overlayed chromatograms of MFA standards and a representative blank matrix sample are shown in Figure S4.

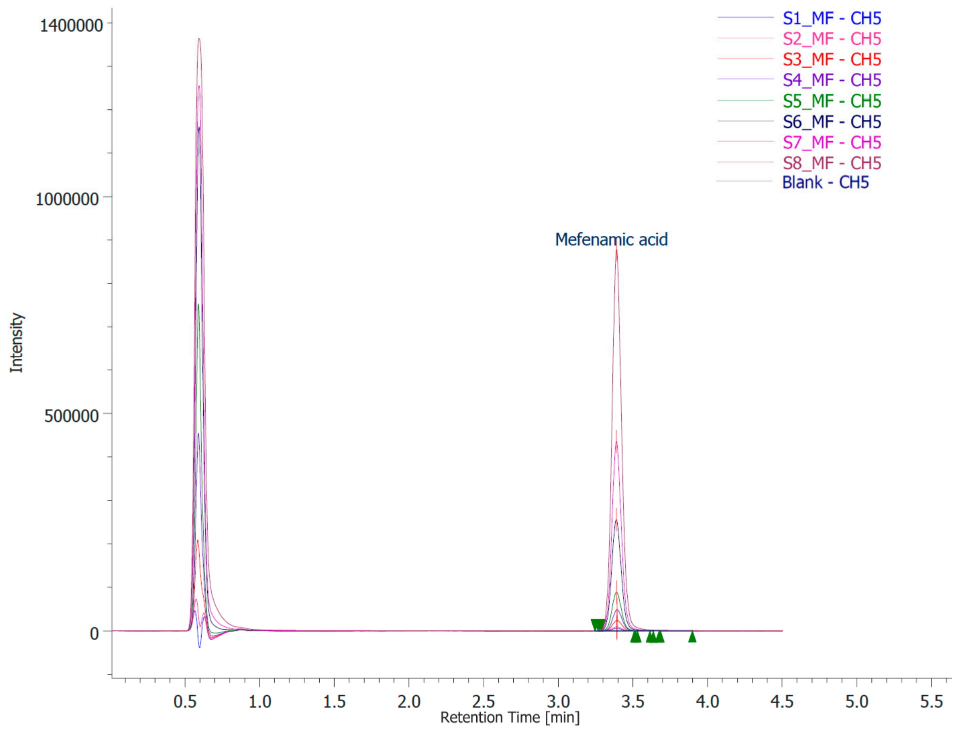


**Figure S4.** Representative overlayed chromatograms of MFA standard samples versus sample matrix blank

### Detection and Quantitation Limits

Based on the S/N ratio, a value on 0.24 µg/mL was calculated as DL, while QL was established at 0.61 µg/mL. Based on the SD of the linear response and slope of the calibration curve, estimated DL was 1.55 µg/mL, while QL was estimated as 4.70 µg/mL.

### Accuracy and Precision

The accuracy and precision (evaluated on two levels: repeatability and intermediate precision) were evaluated using quality control (QC) samples at low (LQC, 15 µg/mL), medium (MQC, 45 µg/mL), and high (HQC, 75 µg/mL) concentration levels. Five replicates for each QC samples were used for the analysis. Mean percent recovery of the analyte in the QC samples 9%) was used to evaluate the accuracy of the method. Precision was estimated in terms of relative standard deviation (RSD, %). The experimental results are depicted in table S4.

**Table S4**. Evaluation of the accuracy and precision of the MFA HPLC method

| | **Repeatability** | | | | **Intermediate precision** | | | |
|---|---|---|---|---|---|---|---|---|
| Sample level | Calculated concentration (µg/mL) | Recovery (%) | Mean recovery (%) | % RSD | Calculated concentration (µg/mL) | Recovery (%) | Mean recovery (%) | % RSD |
| **LQC 15 µg/mL** | 14.99 | 99.96 | 99.70 | 0.30 | 15.18 | 101.20 | 100.15 | 1.06 |
| | 14.97 | 99.80 | | | 15.07 | 100.49 | | |
| | 14.92 | 99.45 | | | 14.89 | 99.30 | | |
| | 14.90 | 99.31 | | | 14.82 | 98.77 | | |
| | 15.00 | 99.98 | | | 15.15 | 100.97 | | |

| | | | | | | | | |
|---|---|---|---|---|---|---|---|---|
| **MQC 45 µg/mL** | 45.03 | 100.08 | 100.88 | 0.63 | 45.17 | 100.39 | 100.70 | 0.92 |
| | 45.44 | 100.99 | | | 45.65 | 101.45 | | |
| | 45.63 | 101.40 | | | 45.63 | 101.40 | | |
| | 45.18 | 100.40 | | | 44.65 | 99.23 | | |
| | 45.70 | 101.55 | | | 45.47 | 101.05 | | |
| **HQC 75 µg/mL** | 75.01 | 100.01 | 100.62 | 0.59 | 75.08 | 100.10 | 100.50 | 0.85 |
| | 76.13 | 101.50 | | | 74.83 | 99.77 | | |
| | 75.18 | 100.24 | | | 76.24 | 101.65 | | |
| | 75.34 | 100.46 | | | 75.87 | 101.16 | | |
| | 75.67 | 100.90 | | | 74.87 | 99.82 | | |

The results demonstrate the high accuracy (with mean recoveries in the 99-101% range) and precision (RSD<1% for repeatability and <1.5% for intermediate precision) of the method for all QC levels.
